# DNA metabarcoding analysis of the bare‐nosed wombat (*Vombatus ursinus*) diet

**DOI:** 10.1002/ece3.11432

**Published:** 2024-05-20

**Authors:** Julie M. Old, Blaire L. Vallin, Rowan K. Thorley, Fiona Casey, Hayley J. Stannard

**Affiliations:** ^1^ School of Science, Hawkesbury Western Sydney University Penrith New South Wales Australia; ^2^ School of Agricultural, Environmental and Veterinary Sciences Wagga Wagga, Charles Sturt University Wagga Wagga New South Wales Australia

**Keywords:** herbivore, ITSF2, marsupial diet, next‐generation sequencing

## Abstract

Wombats are native herbivorous grazers that have adapted to Australia's low‐quality forage. Studies on diet selection by bare‐nosed wombats (*Vombatus ursinus*) are limited and are either observational or based on microhistological studies. The current study determined the diet of wombats through DNA metabarcoding across five study sites in New South Wales over a one‐year period. Metabarcoding was chosen as it is non‐invasive, less time consuming and more specific than traditional techniques. The list of 209 plant species identified as eaten by wombats in this study is much higher than previously reported, with grasses being the most common plant group identified in all samples. Most dietary items identified were introduced plant species. Seasonal differences in plants eaten occurred at four of the five study sites and may reflect dietary abundance and floristic composition at different times of year. Further studies are required to determine if the dietary items differ markedly across the entire range of wombats, and if nutrition influences dietary preferences.

## Graphical abstract

Using metabarcoding we found bare‐nosed wombats (*Vombatus ursinus*) eat mostly grasses, but dietary preferences are significant across season and are habitat specific. Future studies should be investigated across the broader wombat distribution.

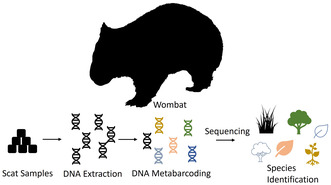

## 1 INTRODUCTION

There are three species of wombat, two hairy‐nosed and the bare‐nosed wombat (*Vombatus ursinus*). The southern hairy‐nosed wombat (*Lasiorhinus latifrons*) is threatened (Woinarski & Burbidge, 2016) whilst the northern hairy‐nosed wombat (*Lasiorhinus krefftii*) is critically endangered (Taggart, Martin, & Horsup, 2016). The bare‐nosed wombat is regarded as Least Concern (Taggart, Martin, & Menkhorst, 2016). All wombats are large native herbivorous grazers, with many adaptations to Australia's low‐productivity habitats (Evans et al., 2006; Johnson, 1998; Sharp & Trusler, 2015). The most obvious and well‐known adaptation is burrowing. Burrowing allows wombats to increase home range size, without needing to increase energy expenditure (Evans et al., 2003, 2006; Hume, 1999). Burrows create a stable microenvironment, allowing for a low metabolic rate to be maintained (Evans et al., 2003, 2006; Hume, 1999). In addition to the benefits of burrowing, wombats have a low daily maintenance energy requirement, with Hume and Barboza (1998) showing wombat energy requirements are less than half the requirements of the koala (*Phascolarctos cinereus*), and one third that of some species of arid‐zone macropods. Wombats also readily digest fibre, using hindgut fermentation and have powerful grinding jaw, resulting in maximum energy extraction from their diet (Barboza, 1993; Evans et al., 2006; Hume, 1989). By optimising energy extraction from food, wombats are able to utilise low‐quality forage in a low production habitat (Green et al., 2015; Hume, 1999). Whilst burrowing benefits wombats, they are also regarded as ecological engineers, with their burrows providing habitat and refuge for other animals, and soil turnover, increasing herbage cover and nitrogen content of soil (Guy & Kirkpatrick, 2021; Old et al., 2018). Wombats emerge from their burrows at night to graze on vegetation. Determining the diet of a species provides useful information for conservation and management plans, as well as providing an understanding of habitat quality, population dynamics and food webs within ecosystems (Dou et al., 2023; Hacker et al., 2021; Williams & Martinez, 2000).

Methods used to determine components of herbivorous diets include direct observation, scat microhistology (Evans et al., 2006; Green et al., 2015, 2017; Matthews, 2010; Rishworth et al., 1995; Woolnough & Johnson, 2000; Woolnough., 1998), stable isotope analysis and stomach content protein electrophoresis (Ando et al., 2018; Espunyes et al., 2019; Iwanowicz et al., 2016). These methods are effective, however in some cases costly, very time consuming, and require expert knowledge. All methods vary in resolution and sampling bias, for example, due to differences in decomposition time and fragmentation of digested plant material (Guo et al., 2018; Iwanowicz et al., 2016; Johnson et al., 1983), microhistology can overestimate the abundance and importance of species with a slower digestion time (Barker, 1986; Iwanowicz et al., 2016). Furthermore, identification to species level may be limited by the similarities between anatomical structures in related genera (Espunyes et al., 2019). Additionally, methods such as stable isotope and stomach content analysis are highly invasive, due to the need to capture and handle animals, and direct observation is hindered in evasive or nocturnal animals in situ.

DNA metabarcoding is a more specific technique than traditional techniques, allowing more accurate identification to the species level, takes less time, and can identify cryptic species without the need for reference plant material (Thongtam na Ayudhaya et al., 2017). It is also not limited by the digestibility or physical attributes of the plant species. Consequently, several species that may not have been identified using microhistology will be detected using DNA metabarcoding (Ando et al., 2018). For example, Rishworth et al. (1995) only identified samples to a vegetation group level (e.g. grass or shrub) rather than to species level, and found 13% of fragments, on average, were unable to be identified in each scat. Additionally, stable isotope analysis has been used previously to determine proportions of grass and browse in the diet of wombats across wide time and space scales (Codron et al., 2011); however, it cannot be used to identify species of plants eaten and must be used in conjunction with another method such as microhistology or DNA metabarcoding.

Faecal DNA metabarcoding is a non‐invasive, high resolution, cost and time‐effective tool frequently used to determine the diet of free‐ranging animals (Ando et al., 2018; Guo et al., 2018; Iwanowicz et al., 2016). The technique has recently been used to investigate the diet of the closely related southern hairy‐nosed wombat (Camp et al., 2020; Sobek & Walker, 2021) and critically endangered northern hairy‐nosed wombat (Casey et al., 2023; Taggart, Martin, & Horsup, 2016). These studies found many more species of plants were included in the diet than previously described using traditional techniques. For southern hairy‐nosed wombats (SHNWs), 15 genera were identified in the diet at one site in Autumn (Sobek & Walker, 2021), whilst for northern hairy‐nosed wombats (NHNWs) a high number of taxa were identified in the diet (191), but most only contributed <0.5% to dietary abundance (Casey et al., 2023).

Studies on the diet of the bare‐nosed wombat (*V. ursinus*) (Shaw 1800), using observation and microhistology techniques (Green, 2005; McIlroy, 1973; Triggs, 1988), have found monocot species, mostly perennial fibrous grasses, dominate the diet (Barboza & Hume, 1992; Evans et al., 2006; Green et al., 2015; Matthews, 2010). Evans et al. (2006) found grasses were the major dietary components of wombats in a eucalypt forest, with up to 97% grasses, mainly *Poa* spp. and *Microlaena stipoides*, 4% sedges and less than 1% forbs, but did not identify any trees or shrubs. Green et al. (2015) likewise found in the Snowy Mountains the bare‐nosed wombat (BNW) diet consists of up to 94% monocot species, with 76.5% *Poaceae* spp., and the remainder other graminoids including Cyperaceae, Junaceae and Liliaceae species, but also found some dicots, mainly shrubs, trees, and forbs. Additionally, Matthews (2010) identified shrubs, flax lily (*Dianella* sp.) and rushes in wombat diets.

Only a few studies identified diet to the species level in wombats due to the lack of specificity in the methods used. Seasonal difference in diet have been studied by Evans et al. (2006); however, this study was limited to three sites. As wombats inhabit a range of bioregions in Australia, and these regions have undergone substantial changes in vegetation structure due to human land use. It is important to understand whether wombats have been able to adapt their diet to these changes and whether changes in vegetation are influencing their health and nutritional status. Furthermore, given their functional significance as ecological engineers, changes in their diet may have wider implications on the Australian landscape.

This study aimed to assess the diet of bare‐nosed wombats, using DNA metabarcoding, to investigate differences in dietary composition between various sites, and determine variations in the percentage composition of native and invasive species within the wombat diet. It is hypothesised that the diet of wombats will exhibit variability across seasons and between different habitats, with potential shifts in the proportion of native and invasive species consumed based on factors such as resource availability, habitat preferences and interspecies interactions.

## 2 MATERIALS AND METHODS

### 2.1 Study sites

Five different study sites were chosen throughout New South Wales (NSW) to account for the wombats' wide distribution throughout eastern and southern Australia. These sites have been utilised in previous studies (Casey et al., 2024; Stannard et al., 2020) and are known to have wombats with and without sarcoptic mange. The sites were the Badger Ground, Coolagolite, Merriwa, Robertson and Wolgan Valley.

#### 2.1.1 ‘Badger ground’ – Rylstone

**FIGURE 1:**
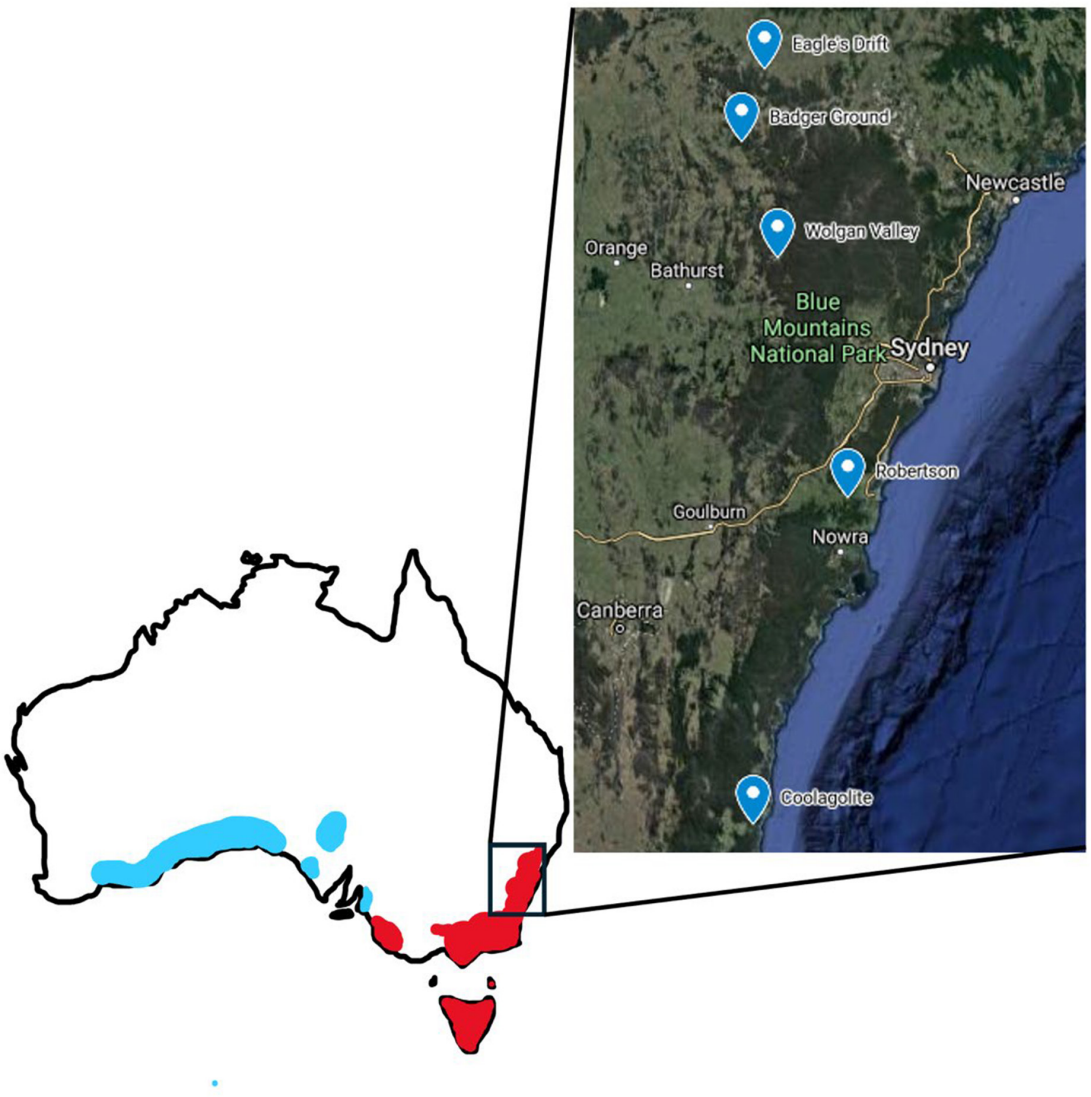
Map of Australia showing the distrubtion of bare‐nosed wombats (red) and southern hairy‐nosed wombats (blue). Inset are the locations of the five study sites where BNW scats were collected for dietary analysis. Adapted from Taggart, Martin, and Menkhorst (2016) and Woinarski and Burbidge (2016).

‘Badger Ground’ (32°38′ S, 149°58′ E) is located in Breakfast Creek, NSW, approximately 20 km from the nearest town Rylstone and approximately 115 km north of Lithgow, NSW (Figure 1). The property is privately owned and consists of 330 ha of dry sclerophyll forest, grassy box woodland and open grassland. The property also runs adjacent to a gully rainforest with natural springs. The current owners of the property have converted the land from logging, livestock grazing (sheep and cattle) and a large‐scale market garden, to a protected area through a Conservation Agreement with NSW National Parks and Wildlife Service. Part of this agreement includes feral and pest animal and noxious weed management. Supplementary feeding of the local wombat population occurs during low rainfall seasons with lucerne hay (*Medicago sativa*) and native grasses collected from roadside greenspace (Pridmore, S., personal communication, 2019). The area has an mean annual rainfall of 670 mm and an mean temperature range of 8–16°C (Bureau of Meteorology, 2019).

#### 2.1.2 Coolagolite

The study site in Coolagolite is a privately owned property, approximately 5 km north east of the town of Coolagolite, NSW (36°23′ S, 150°00′ E) (Figure 1). Coolagolite is within the Bega Valley Shire, an area dominated by wet and dry forests. The study site is mostly cleared riparian zone, surrounded by dry forest and dry rainforest. Narira Creek runs adjacent to the property. Part of the study site is under a Voluntary Conservation Agreement with NSW National Parks and Wildlife Service. Due to this agreement, the property owners actively manage noxious weeds, feral animals and manage the land in a way that promotes increased native biodiversity. On average, this area has a mean rainfall of 917 mm and mean temperature range of 12–20°C (Bureau of Meteorology, 2019).

#### 2.1.3 ‘Eagle drift’ – Merriwa

Eagles Drift (32°11′ S, 150°05′ E) is located approximately 30 km from the town of Merriwa, NSW and approximately 210 km west of Newcastle, NSW (Figure 1). The privately owned property is approximately 577 ha and consists of dry sclerophyll forest, box woodland and open pasture. Previously the property was used for sheep and cattle grazing, wheat (*Triticum* spp.) and barley (*Hordeum vulgare*) production, and bush rock collection. Currently, there is a small hobby cattle farm being run on the property. There is a current Conservation Agreement with the Nature Conservation Trust for 270 ha of the property, which has removed cattle grazing in this area allowing native vegetation regeneration. On average, this area has an annual rainfall of 600 mm and mean annual temperature range of 9–24°C (Bureau of Meteorology, 2019).

#### 2.1.4 Robertson

The site in Robertson is a privately owned property (−34°35′19.215″ S, 150°35′28.932″ E) located 8 km east of the town of Robertson, NSW (Figure 1). The site is approximately 35 km from the coast and is approximately 5 km north of the Budderoo National Park. The majority of the land is utilised as sheep grazing and is mostly open grassland, with small amounts of remnant box woodland surrounding the property. Robertson is located with the southern highlands of NSW, which is dominated by shale forest and woodland, with large areas of cleared grassland for large animal grazing. This area has a mean annual rainfall of 701 mm and mean annual temperature range of 8–19°C (Bureau of Meteorology, 2019).

#### 2.1.5 Wolgan Valley

Wolgan Valley is approximately 35 km north of Lithgow NSW and is situated next to the Greater Blue Mountains World Heritage area. The study site is located within the Emirates One&Only Wolgan Valley Eco Resort (33°15′ S, 150°10′ E) (Figure 1). This site was previously a cattle station but has been managed as a luxury ecotourism resort for over a decade. The resort also maintains several conservation programs, including feral animal control, fauna surveys and habitat regeneration. The site is dominated by open grassland surrounded by dry sclerophyll forest and remnant regrowth. The site also has two water ways, the Wolgan River and Carne Creek, which run through the site and have minimal vegetation on the riverbanks in riparian areas, which has led to erosion. The area has a mean annual rainfall of 758 mm and mean temperature range from 5 to 25°C (Bureau of Meteorology, 2019).

### 2.2 Sample collection

Scat samples (112) were collected seasonally in Jun‐Aug 2018, Oct/Nov 2018, Feb 2019 and Apr 2019. A minimum of six fresh scats, identified by their colour and consistency, were collected from each of the five sites and placed in zip lock bags on ice as per Old et al. (2020). The date and GPS location of scat collection were recorded. Once returned to the laboratory, each scat sample, made up of pellets, was mixed to break up the pellets and to ensure the samples were homogeneous, prior to a subsample of approximately 2 g being placed into a labelled test tube and stored at −20°C until analysis.

### 2.3 DNA extraction and metabarcoding

DNA extraction and metabarcoding was performed using Next Gen Sequencing by Australian Genomic Research Facility, Adelaide (AGRF). DNA was extracted from 112 scats using the Qiagen DNA Stool Mini Kit (Qiagen, Hilden, Germany) using the manufacturer's guidelines. Of the 112 samples extracted, 92 had DNA of sufficient quality based on spectrophotometry, for PCR, and included 19 from Badger Ground, 19 from Coolagolite, 14 from Merriwa, 20 from Robertson and 20 from Wolgan Valley (Table 1). The PCR conditions and protocols, including the primers (ITSF2 TGTGAATTGCARRATYCMG; ITS2R CCCGHYTGAYYTGRGGTCDC), were based on Moorhouse‐Gann et al. (2018) to amplify a fragment 187–387 bp in length, and included the use of sterile water as a negative control. The 92 sequences were then analysed on an Illumina MiSeq platform. Metabarcoding involved image analysis through MiSeq Control Software (MCS) v2.6.2.1 and Real Time Analysis (RTA) v1.18.54, followed by sequence data generation using the Illumina bcl2fastq 2.20.0.422 pipeline. Bioinformatic analysis performed by AGRF involved quality control, operational taxonomic unit (OTU) clustering and taxonomic classification.

**TABLE 1:** Number of scats samples per site and season that had DNA of high enough quality for sequencing.

Site	Autumn	Spring	Summer	Winter	Total
Badger Ground	4	5	4	6	19
Coolagolite	5	5	4	5	19
Merriwa	3	3	4	4	14
Robertson	4	7	3	6	20
Wolgan Valley	5	4	7	4	20
Total	21	24	22	25	92

Paired‐end reads were aligned with the forward and reverse reads using PEAR (version 0.9.5) and primers trimmed. Trimmed sequences were processed using Quantitative Insights into Microbial Ecology (QIIME 1.8) (Caporaso et al., 2010) 4 USEARCH2,3 (version 8.0.1623) and UPARSE software (Edgar, 2013). Sequences were then quality filtered using USEARCH tools and sequences were sorted by abundance. Unique reads were discarded (unpaired reads), and sequences were clustered. To obtain number of reads in each OTU, reads were mapped back to OTUs with a minimum identity of 97% in Genbank. Taxonomy was assigned using NCBI blast.

### 2.4 Data analysis

Only plant species were included in the analysis, hence sequences were filtered to remove species not identified as plants (e.g. bacteria, protozoa) or those having <100 reads assigned OTUs. Species were categorised by clade (monocot, eudicot or Gymnospermae) and origin (introduced or native). A further three sequences were unable to be identified to species level and were included in the analysis despite only being identified to genera level.

Relative read abundance (RAA) was used as a proxy for abundance of taxa within the diet, which is a measure of the percentage of total reads a taxon contributes per sample (Deagle et al., 2019), hence in our study scats. Frequency of occurrence (FOO), the percentage of scat samples in which a taxon was identified, was also used as a measure of taxa occurrence within the diet. RAA and FOO were used to provide a measure of dietary item composition within scats and across all scats, respectively. Using Primer 7 software (PRIMER, 2023) a PERMANOVA was used to determine if there was a significant difference in the abundance of each taxon in the diet across sites and seasons. A zero‐adjusted Bray–Curtis dissimilarity index was calculated for each site, and an ANOSIM (analysis of similarities) was used to determine whether the diet varied between seasons at each site, with 9999 permutations of the test statistic. SIMPER (similarity percentages) was used to determine the average dissimilarity between seasons at each site and each taxa contribution (%) to dissimilarity (Clark & Warwick, 2001). The Shannon diversity index (*H*) was calculated from species richness relative to abundance $H^{\prime }=-\sum_{i=1}^{s}p_{i}\;\ln\;p_{i}$ where *H*′ is the species diversity index, *s* is the number of species, and *p*_i_ is the proportion of individuals of each species belonging to the ith species of the total number of individuals.

## 3 RESULTS

A total of 92 scats had DNA successfully extracted and sequenced, 19 from Badger Ground, 19 from Coolagolite, 14 from Merriwa, 20 from Robertson and 20 from Wolgan Valley (Table 1). Overall, three sequences could only be identified to plant Genus level, and 209 were identified to species level, in the scats, with all assumed to be consumed by wombats. Most of the plants identified in the scats were monocots, followed by eudicot species (Table 2). Most plants identified in the scats belonged to the Poaceae Family (55.2%) followed by the Asteraceae (11.3%) and Fabaceae (6.6%) Families. Of those plants identified to the species level 73.4% were introduced species and 26.6% were native species (Table S1). The highest number of species identified in the diet of wombats was at Badger Ground and the lowest number was at Wolgan Valley (Table 2). One species of Gymnospermae was identified in the diet at three sites, *Pinus contorta* (lodgepole pine). Between 0.26% and 2.11% of DNA sequences were unidentified depending on site (Table S1).

**TABLE 2:** Number of plant species present in each Clade identified in the diet at each site.

	Badger ground	Coolagolite	Merriwa	Robertson	Wolgan Valley
Monocots	85	89	75	85	78
Eudicots	67	44	44	50	32
Gymnospermae	1	1	0	1	0
Total	153	134	119	136	110
Shannon Index (*H*)	3.37	2.48	3.00	2.27	2.23

### 3.1 Relative abundance and frequency of occurrence

**FIGURE 2:**
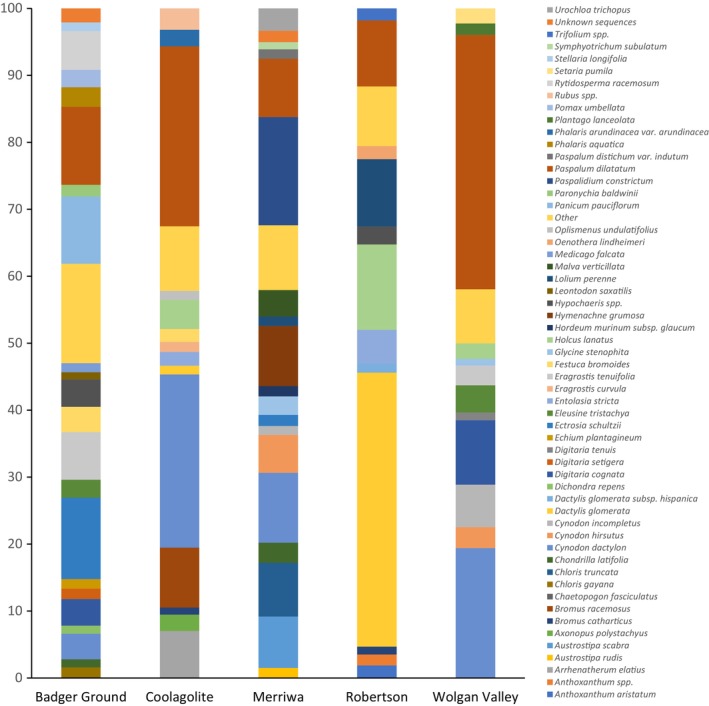
Relative abundance of plants in the diet of bare‐nosed wombats at five sites across New South Wales. Plants that had <1.0% relative abundance were added together and included as ‘other’.

In terms of relative abundance, grasses were the most consumed dietary item. The species with the highest RAA at each site were *Ectrosia schultzii* (hare's foot grass) at Badger Ground, 12.2%; *Paspalum dilatatum* (dallis grass) at Coolagolite, 26.9% and Wolgan Valley, 38.0%; *Paspalum constrictum* (knottybutt grass) at Merriwa, 16.2% and *Dactylis glomerata* (orchard grass) at Robertson, 40.9% (Figure 2). *P. dilatatum* contributed >8.7% of the RRA in the diet at all five sites and *Cynodon dactylon* (Bermuda grass) >3.8% at all sites except at Robertson where it was only 0.3% (Table S1).

For FOO of plant species in the scats at each site, both Badger Ground and Coolagolite had nine species of plant found in 100% of scats, Merriwa, and Robertson both had 10 species found in 100% of scats, and Wolgan Valley had only one species found in 100% of scats. *P. dilatatum* was found in 100% of scats across all five sites (Table S2).

### 3.2 Site differences

Of the 209 plant species identified, 39 were found in the diet of wombats at all five sites (Table S1). There were 24 species that were unique to a specific site. Badger Ground had eight species of plant unique to that site while Coolagolite had five, Merriwa had six species, Robertson had five species and Wolgan Valley had no unique species identified. At all sites most species identified in the scats were introduced plant species, Badger Ground 67.1%, Coolagolite 76.9%, Merriwa 67.0%, Robertson 74.4% and Wolgan Valley 70.8%.

A PERMANOVA showed there was a significant difference between sites (*F*_4,72_ = 17.09, *p* < .05) and a significant interaction between site and season (*F*_12,72_ = 2.62, *p* < .05) for the relative abundance of plants in the diet. The average dissimilarity was highest for Badger Ground versus Robertson (96.51%) and lowest for Coolagolite versus Wolgan Valley (65.62%). The species *C. dactylon*, *D. glomerata*, *Holcus lanatus*, *P. dilatatum*, and *Paspalidium constrictum* had the highest contribution to dissimilarity across the sites (Table S3).

### 3.3 Seasonal differences

**FIGURE 3:**
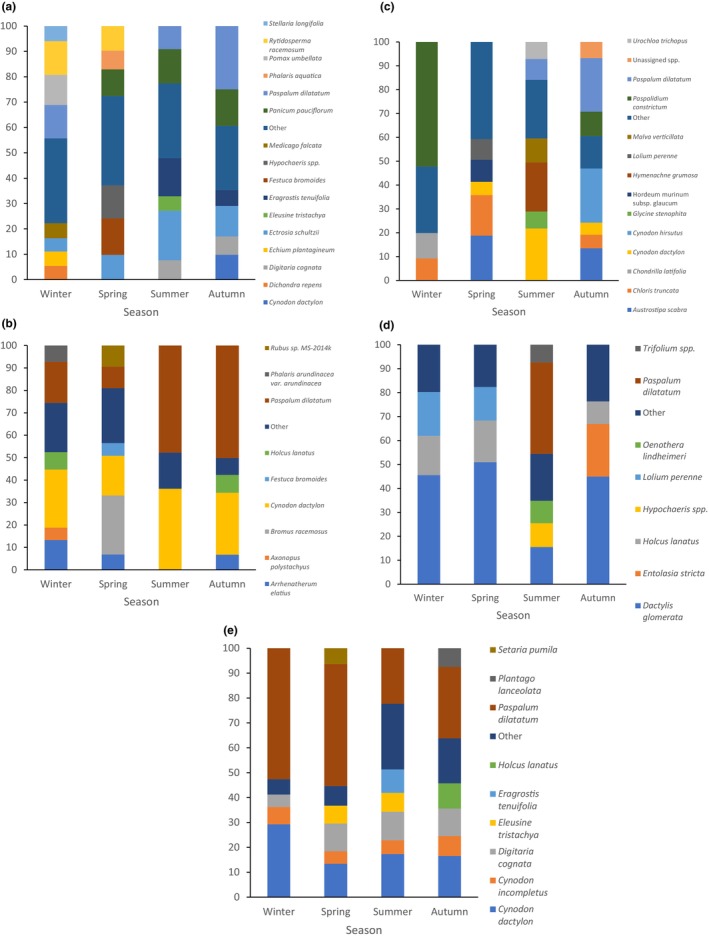
Seasonal relative abundance of plants in the diet of bare‐nosed wombats at five sites across New South Wales. Plants that had <5.0% relative abundance were added together and included as ‘other’, (a) Badger Ground, (b) Coolagolite, (c) Merriwa, (d) Robertson, (e) Wolgan Valley.

A PERMANOVA showed there was a significant difference in the relative abundance of plants in the diet seasonally (*F*_3,72_ = 3.32, *p* < .05). A one‐way ANOSIM for each site showed that there were significant differences between seasons at all sites except Wolgan Valley (*R* ^2^ = −.042, *p* = .64; Figure 3e). Pairwise tests showed that at Badger Ground there were significant differences between autumn and winter (*R* ^2^ = .433, *p* < .05), autumn and spring (*R* ^2^ = .581, *p* < .05), winter and spring (*R* ^2^ = .531, *p* < .05), winter and summer (*R* ^2^ = .571, *p* < .05) and summer and spring (*R* ^2^ = .838, *p* < .05). At Coolagolite there were significant differences between autumn and winter (*R* ^2^ = .212, *p* < .05), autumn and spring (*R* ^2^ = .496, *p* < .05), winter and spring (*R* ^2^ = .300, *p* < .05), winter and summer (*R* ^2^ = .331, *p* < .05) and summer and spring (*R* ^2^ = .513, *p* < .05). There was was no significant difference between autumn and summer at both sites (Badger Ground *R* ^2^ = −.115, *p* = .86 and Coolagolite *R* ^2^ = −.006, *p* = .44). At Badger Ground, the average dissimilarity was highest for autumn versus winter, 89.74% with the highest contribution from the species *Panicum pauciflorum* followed by *P. dilatatum* (Table S4; Figure 3a). At Coolagolite the average dissimilarity was highest for autumn versus spring, 72.48% with the highest contributing species being *P. dilatatum* followed by *Bromus racemosus* (smooth brome) (Table S5; Figure 3b). Pairwise tests showed that at Merriwa there were significant seasonal differences between autumn and summer (*R* ^2^ = .741, *p* < .05), winter and summer (*R* ^2^ = .906, *p* < .05) and summer and spring (*R* ^2^ = .741, *p* < .05). There were no significant differences between winter and spring (*R* ^2^ = .593, *p* = .06), autumn and winter (*R* ^2^ = .463, *p* = .09) or autumn and spring (*R* ^2^ = .519, *p* = .10). The average dissimilarity was highest for winter versus summer, 93.80% with the highest contribution from the species *P. constrictum* followed by *C. dactylon*, being higher in winter and summer, respectively (Table S6; Figure 3c). At Robertson, there were significant seasonal differences between winter and summer (*R* ^2^ = .759, *p* < .05) and summer and spring (*R* ^2^ = .742, *p* < .05), with a higher portion of *D. glomerata* present in winter and spring, respectively. There were no significant differences between autumn and summer (*R* ^2^ = .426, *p* = .11), winter and spring (*R* ^2^ = −.038, *p* = .58), autumn and winter (*R* ^2^ = .202, *p* = .13) and autumn and spring (*R* ^2^ = .177, *p* = .12). The average dissimilarity was highest for spring versus summer, 69.35% with the highest contribution from the species *P. dilatatum* followed by *D. glomerata* (Table S7; Figure 3d).

## 4 DISCUSSION

Using metabarcoding this study identified 209, mostly introduced (73%), monocot and eudicot species, and three sequences identified to genera, in 92 wombat scat samples across the five sites investigated. The number of plant species identified in this study in scats was substantially more than previously reported. From studies that identified plants to the species level between 15 and 63 species have been identified previously as wombat food items (Table 3) (Evans et al., 2006; Green et al., 2015; Mallett & Cooke, 1986). Only one of the top five species identified by Green et al. (2015) was identified in our study at one site, *Festuca rubra*, 0.22% RAA at Robertson. Three of the thirty‐five species identified as wombat food by Evans et al. (2006) were identified in our study, *Lolium rigidum*, *D. glomerata* and *H. lanatus* (Table 3)*. Lolium rigidum* was only 0.02% RRA at one site, while *D. glomerata* (most eaten species at Robertson) and *H. lanatus* were found in the diets at four of the five sites in varying abundance, and all are introduced plants species. *Lolium perenne* was identified in the diet at all five of our sites and has previously been identified in wombat diets in South Australia (Table 3) (Mallett & Cooke, 1986). The differences in plant species identified in wombat scats may reflect differences in floristic occurrence and abundance and site differences.

**TABLE 3:** Diet of bare‐nosed wombats recorded previously.

Study	Green et al. (2015) – Comparison of wombat diet at sites before and after fire	Evans et al. (2006)	Matthews (2010) – Observations of wombat diet in snow	Davis et al. (2008) – Compared dietary overlap between wombats and kangaroos	Rishworth et al. (1995)	Mallett and Cooke (1986)
Method	Microhistology	Microhistology	Microhistology	Microhistology of stomach samples	Microhistology	Microhistology
Site	Snowy Mountains, NSW	Riamukka State Forest, NSW	Snowy Mountains, NSW	Yanakie Isthmus, Victoria	Buccleuch State Forest, NSW	Messent Conservation Park and Coorong National Park, SA
Number of scats/stomach samples	111 scats	80 scats	34 scats	20 stomach samples	28 scats	13 scats
Period of collection	Immediately after fire in 2003, medium term after fire in 2007 and the long term after fire 2012	Seasonally over 2 years	July–August 2008	Jun 2004–Feb 2005	May 1990	Jan, Apr, Jul, Oct 1985
Number of plant species identified	63	>35				15
Summary of most common species eaten	Top species in terms of mean abundance: *Poa fawcettiae*, *Poa costiniana*, *Poa hiemata*, *Dianella tasmanica*, *Festuca rubra*	Most abundant in samples identified to species level: *Microlaena stipoides*, *Danthonia racemose*, *Lolium rigidum*, *Dactylis glomerata*, *Holcus lanatus*, *Themeda australis*	Food items observed to be eaten included grasses of *Poa* spp. (62% of observations), other grasses (15.5%), shrubs (13.2%); predominantly dusty daisy bush (*Olearia phlogopappa*), as well as leafy bossiaea (*Bossiaea foliosa*), and cascade everlasting (*Ozothamnus secundiflorus*), flaxlily (*Dianellata smanica*; 1.6%) and rushes (0.8%) Scat analysis confirmed monocots made up the majority of the wombat diet. Some wombats consumed up to 26% dicots, and more dicots were consumed when foraging in snow depths greater than 30 cm	Mostly monocots (>11 times dicots), particularly grasses (>50%) and grass‐like plants (>20%)	Grasses were the dominant food group. Fragments of roots were found in every faecal sample. Bark constituted 2.2%–10.1%	Grasses: *Argostis billardieri*, *Danthonia setacea*, *Distichlis distichophylla*, *Ehrharta calycina*, *Lolium peenne*, *Stipa stipoides* Sedges: *Baumea juncea*, *Gahnia trifida*, *Hypolaena fastigiate*, *Isolepis nodosa*, *Lepidosperma semiteres*, *Lepidosperma viscidum*, *Tetraria capillaris* Herbs: *Lomandra effuse*, *Trifolium fragiferum*

The most common plant group identified in all samples were grasses, as reported previously (Evans et al., 2006; Green et al., 2015, 2017; Matthews, 2010; Rishworth et al., 1995). Interestingly, when compared to the closely related NHNW, with 90% of their diet including grass (Casey et al., 2023), the diet of the BNW contains a much lower abundance of grass overall (55% of all species identified in the diet). This difference between species may be a result of species or habitat differences. The most common grass species identified in our study in the diet of the BNW was *P. dilatatum* (>8.7% RAA at all sites)*. P. dilatatum* has previously been found to be negatively selected by BNWs (Evans et al., 2006) and was not identified in other studies of BNW diets (Green et al., 2015, 2017). In our study, *P. dilatatum* appears to be an important dietary item and particularly at Wolgan Valley, with abundance being 37.9%. *D. glomerate* has previously been identified as an important species in the wombat diet (Evans et al., 2006) and was also identified as one of the most abundant species in this study at Robertson (41%). Despite *Poa* spp. being identified as an abundant species in the diet previously (Evans et al., 2006; Green et al., 2015; Matthews, 2010), this was not the case in our study. Likewise, despite monocots being the dominant clade identified in scat samples (56%–71%), they were not as abundant as in previous studies. Matthews (2010) found monocots made up to 93.3% of the diet, Green (2005) stated monocots were consistently at least 85% of the wombat diet, and Green et al. (2015) suggested between 89% and 94% of the wombat diet was made up of monocots. It is possible the variation in abundance from previous studies is due to diet selectivity, as Rishworth et al. (1995) showed eudicots were consistently preferred less by wombats and Evans et al. (2006) showed that wombats selected monocot species over eudicot species. Additionally, the average percentage of eudicots in this study (29%–44%) was higher than previously seen. Matthews (2010) recorded an average of 6.7% dicots and Green et al. (2015) found between 6% and 10% dicots in the diet of wombats. However, Matthews (2010) did find the proportion of eudicot plants in an individual's diet would vary, with samples consisting of up to 26% eudicot species. It is possible, due to the high resolution of DNA metabarcoding, more eudicot species were detected, which may account for the increase in eudicot species identified in our study. It is also possible that some plant species identified using DNA metabarcoding in scat samples are not dietary items of wombats and may have occurred as a result of contamination of the scat sample once deposited on the ground. Future studies could extract scat samples directly from wombats to reduce potential contamination; however, this would involve invasive techniques, which this study aimed to reduce.

The current study identified one Gymnospermae spp., *P. contorta*. While previous studies have identified species outside of monocots and eudicots in the diet of wombats, Gymnospermae have not previously been separated from monocot and eudicot species during analysis. Due to their low occurrence, studies often analysed species abundance, or plant type (e.g. grass or forb), rather than conducting analysis based on clades identified. Rishworth et al. (1995) identified pine needles in wombat scats collected within pine plantations, indicating that they likely eat pine when available.

Wombat diets at different sites differed significantly with 39 species found at all sites and 24 unique to a specific site. Variations in diet of the NHNW and SHNW at different sites have also been observed (Camp et al., 2020; Casey et al., 2023). The site differences are likely reflected in the dietary availability and floristic abundance, as observed for SHNWs (Camp et al., 2020), hence likely explain the difference in diet at the different sites; however, our study would have benefited from a floristic abundance assessment conducted at the same time. The current study was also conducted during a drought, which may also have impacted plant species available to wombats, given lower annual rainfall and higher than average temperatures (Bureau of Meteorology, 2019). At all sites, introduced species made up a higher portion of the diet compared to native species. This occurrence of introduced species were likely representative of the floristic composition of the habitats, as often introduced species out compete native grasses and sequester resources (Grice et al., 2013; Groves et al., 2003). Although weeds are actively managed at Coolagolite, this site had the highest proportion of introduced plant species identified in the scats; however, not all introduced species are being controlled at this site.

Seasonal differences in the diet were also evident, except for one site (Wolgan Valley), and has been reported by Casey et al. (2023) for the NHNW. These seasonal dietary differences are likely to reflect the dietary abundance and floristic composition at different times of year, as indicated by the limited study on SHNWs during late summer/early Autumn by Camp et al. (2020). Seasonal differences in diet selection may also relate to the changes in nutrient availability within the habitats (Casey et al., 2024). There is less variety of plant species at the Wolgan Valley site and consistency in availability of the species available across seasons, which would account for a lack of significant difference across seasons at this site.

Our study found most plant species identified in the diet of BNWs were introduced species (55%). This is in contrast to Davis et al. (2008) who found BNWs ate more native than exotic species based on a study investigating stomach content analysis. It is possible that this variation may be due to the classification of what is an native and exotic species, as Davis et al. (2008) does not provide a definition for what was considered native and introduced, or a full list of the plants consumed by wombats. When compared to hairy‐nosed wombats, the percentage of introduced and native plant species in the diet were approximately 55%–58% and 42%–45%, respectively, for the NHNW, with introduced buffel grass the dominant species (Casey et al., 2023). Whilst Camp et al. (2020) only sampled in February to March 2013 they found the majority of species eaten were introduced (12 of the 16 plant species found at all three sites).

Since European settlement, the habitat of BNWs has changed significantly (reviewed in Thorley and Old (2020)), and there has been an abundance of grasses and other plants introduced into the environment. Wombats, and all native herbivores have adapted to the changes in floristic composition in their habitat, however it is likely that these changes in the floristic communities have been both beneficial and disadvantageous (McArthur et al., 2014; Pyšek et al., 2012). For example, changes in availability of dietary items and floristic composition for NHNWs, specifically increases in introduced buffel grass (*Cenchrus ciliaris*) in the NHNWs habitat and has led to significant changes in the dietary abundance and FOO for the species over time (Casey et al., 2023). Similar changes have also appeared to have occurred for SHNWs, whereby the native plants have been largely replaced by introduced species in the environment (Camp et al., 2020; Sobek & Walker, 2021). Camp et al. (2020) found the diet of the SHNW included toxic plant species during times of food shortages, with one site (Portee) having introduced *Brassica tournefortii* and *Carrichtera annua* prominently featured in the scats and most animals at this site showing signs of glucosinolate poisoning. Nutritional studies would assist in understanding the role these floristic changes have on nutrient intake and health of wombats. Furthermore, characterising the diet of BNWs over a longer timeframe, including during extremes of droughts and in very wet years would likely provide more insights into how introduced and endemic species influence diet selection in BNWs, because of expected changes in floristic community changes during these climate extremes.

Some unexpected findings occurred in our study. The first was that our study is the only study to identify *Bromus* spp. in the BNW diet and directly contradicts Evans et al. (2006) who suggested it was not eaten by wombats. One further very unexpected finding in this study was that one common introduced grass species, kikuyu (*Cenchrus clandestinus*), was not detected in any of the scat samples collected, despite being abundant in at least some of the sites (unpublished data). We would have expected BNWs to consume this commonly available grass and are aware in at least some cases, wombats will eat this grass when provided with it in captivity and may be seasonally specific (A. Cox and K. Schweth, personal communication). On further investigation we directly searched the NCBI database for the kikuyu ITS DNA sequence and found that whilst the genome for kikuyu was a recent addition to the database, there is no annotated ITS sequence for kikuyu available. Hence, we cannot determine if the BNWs in this study did consume kikuyu as a component of their diet, or if it was simply not detected because of a lack of sequence in the database. This issue highlights one of the limitations of using DNA metabarcoding for assessing diet. Presumably, this is not the only plant species that currently does not have an ITS sequence available in the database, that is present in the BNW habitat. Despite a lack of this sequence, and presumably other sequences being currently available in the database, over time additional sequences will be added to the database and hence, reanalysis of the sequences, particularly the 2.11% not identified at one site (Badger Ground) in this study, are likely to provide additional insights. Furthermore, while reassessing the data from this study in a future study, will be possible, this is not the case for most traditional dietary analysis techniques, such as microhistology.

This study presents a comprehensive analysis of the BNW diet across five localities in NSW using DNA metabarcoding. The study identified 209 plant species were eaten by BNWs at these five sites. Our study has shown significant differences in wombat diets across study sites and significant seasonal changes across four of the five study sites. We found wombats consumed mostly introduced grasses. The dietary items consumed by BNWs are likely a reflection on the abundance and floristic complsotion at different times of the year at the different sites. However, given the wide distribution of BNWs (Thorley & Old, 2020) this study should be extended to include a larger number of sites and would likely provide a better reflection of the dietary differences of BNWs throughout their broader range. Furthermore, while the current study provides an extensive list of species eaten by bare‐nosed wombats, future studies could conduct a floristic study at the same time to determine the influence of season on the availablity of dietary items in the habitat.

New metabarcoding techniques are significantly less labour intensive than techniques used previously to assess BNW diet, allowing for a larger number of wombat scat samples to be analysed from a larger number of sites, and a seasonal comparison made. Furthermore, metabarcoding is more sensitive than previously used techniques, allowing a much larger number of plant species to be identifed in the diet of the BNW. The plant species consumed by BNWs included mostly introduced plant species suggesting that wombats can readily adapt to floristic changes in their habitat. Thus, expected changes in the floristic community in wombat habitats as a result of climate change are unlikely to greatly impact wombats. This study also suggests that captive wombats can be fed a wide and varied plant‐based diet, including introduced species, but it must contain a high volume of grasses.

## AUTHOR CONTRIBUTIONS

**Julie M. Old:** Conceptualization (equal); data curation (equal); formal analysis (equal); funding acquisition (equal); methodology (equal); project administration (equal); supervision (equal); writing – review and editing (equal). **Blaire L. Vallin:** Investigation (equal); writing – original draft (equal). **Rowan K. Thorley:** Formal analysis (equal); writing – original draft (equal). **Fiona Casey:** Formal analysis (equal); visualization (equal). **Hayley J. Stannard:** Conceptualization (equal); data curation (equal); formal analysis (equal); funding acquisition (equal); methodology (equal); project administration (equal); supervision (equal); writing – review and editing (equal).

## FUNDING INFORMATION

The project was funded by a Charles Perkins Centre EMCR grant awarded to HJS and the Wildlife Disease Association Challenge Grant (Experiment.com) crowd funding awarded to JMO and HJS.

## CONFLICT OF INTEREST STATEMENT

On behalf of all authors, the corresponding author states that there is no conflict of interest.

## Supporting information


Tables S1–S7.

## Data Availability

The data that support this study are available in the article and accompanying online supplementary material.
