# Supplementary material for: DNA metabarcoding analysis of the bare‐nosed wombat (Vombatus ursinus) diet
Source: Ecol Evol. 2024 May 20;14(5):e11432. doi: 10.1002/ece3.11432 (PMC11103767; doi:10.1002/ece3.11432)
Supplement: Supplementary file 1 — Tables S1–S7. [file ECE3-14-e11432-s001.docx]

**Appendix**

Table S1. Relative read abundance (%) of plants identified in scats of bare-nosed wombats from five sites in New South Wales

| Scientific name | Common names | Clade | Family | Origin | Badger Ground | Coolagolite | Merriwa | Robertson | Wolgan Valley |
| --- | --- | --- | --- | --- | --- | --- | --- | --- | --- |
| *Acaena caesiglauca* |  | eudicots | Rosaceae | I | 0.0809 | 0.0000 | 0.0000 | 0.0000 | 0.0000 |
| *Agrostis castellana* |  | monocots | Poaceae | I | 0.0000 | 0.0039 | 0.0000 | 0.0459 | 0.0000 |
| *Agrostis mertensii* |  | monocots | Poaceae | I | 0.0001 | 0.6108 | 0.0000 | 0.2718 | 0.0974 |
| *Anagallis arvensis* | Scarlet pimpernel, red pimpernel, red chickweed, poor man's barometer, poor man's weather-glass, shepherd's weather glass or shepherd's clock | eudicots | Primulaceae | I | 0.0000 | 0.0291 | 0.0000 | 0.0000 | 0.0000 |
| *Angophora costata* | Sydney red gum, rusty gum or smooth-barked apple | eudicots | Myrtaceae | N | 0.0363 | 0.0000 | 0.0000 | 0.0000 | 0.0000 |
| *Anthosachne aprica* |  | monocots | Poaceae | I | 0.0072 | 0.0000 | 0.0000 | 0.0000 | 0.0043 |
| *Anthoxanthum alpinum* | Sweet vernal grass | monocots | Poaceae | I | 0.0000 | 0.0010 | 0.0000 | 0.0628 | 0.0000 |
| *Anthoxanthum aristatum* | Awned vernalgrass; annual vernalgrass | monocots | Poaceae | I | 0.0000 | 0.7719 | 0.0010 | 1.8706 | 0.0004 |
| *Anthoxanthum odoratum* | Sweet vernal grass | monocots | Poaceae | I | 0.0000 | 0.0426 | 0.0000 | 0.0740 | 0.0000 |
| *Anthoxanthum ovatum* | Small sweet grass | monocots | Poaceae | I | 0.0000 | 0.0003 | 0.0000 | 0.0170 | 0.0000 |
| *Anthoxanthum sp. PT-2016* |  | monocots | Poaceae | I | 0.0001 | 0.3254 | 0.0005 | 1.6284 | 0.0012 |
| *Arrhenatherum elatius* | Bulbous oat grass; false oat-grass; tall oat-grass; tall meadow oat; onion couch; tuber oat-grass | monocots | Poaceae | I | 0.0000 | 7.0204 | 0.0009 | 0.1178 | 0.0075 |
| *Austrostipa rudis* |  | monocots | Poaceae | N | 0.0117 | 0.0033 | 1.4920 | 0.0008 | 0.0388 |
| *Austrostipa rudis subsp. nervosa* |  | monocots | Poaceae | N | 0.0030 | 0.0012 | 0.2454 | 0.0001 | 0.0035 |
| *Austrostipa scabra* | Rough speargrass | monocots | Poaceae | N | 0.4313 | 0.0001 | 7.6617 | 0.0038 | 0.6237 |
| *Austrostipa scabra subsp. Falcata* |  | monocots | Poaceae | N | 0.0287 | 0.0002 | 0.0721 | 0.0000 | 0.0001 |
| *Austrostipa trichophylla* |  | monocots | Poaceae | N | 0.0009 | 0.0000 | 0.0219 | 0.0000 | 0.0001 |
| *Austrostipa verticillata* | Slender Bamboo grass | monocots | Poaceae | N | 0.0001 | 0.0000 | 0.6359 | 0.0002 | 0.3275 |
| *Avena sativa* | Oats | monocots | Poaceae | I | 0.1845 | 0.0455 | 0.0005 | 0.1716 | 0.0000 |
| *Axonopus argentinus* |  | monocots | Poaceae | I | 0.0000 | 0.0100 | 0.0000 | 0.0002 | 0.0000 |
| *Axonopus polystachyus* |  | monocots | Poaceae | I | 0.0001 | 2.4505 | 0.0019 | 0.9368 | 0.0872 |
| *Boerhavia erecta* | Erect spiderling; the erect boerhavia | eudicots | Nyctaginaceae | I | 0.0000 | 0.0000 | 0.1475 | 0.0000 | 0.0000 |
| *Bothriochloa ischaemum* |  | monocots | Poaceae | I | 0.0325 | 0.0000 | 0.0238 | 0.0000 | 0.2538 |
| *Brachiaria subquadripara* |  | monocots | Poaceae | I | 0.0039 | 0.0000 | 0.3100 | 0.0001 | 0.0291 |
| *Briza minor* | Little quaking grass | monocots | Poaceae | I | 0.0138 | 0.0000 | 0.0000 | 0.0002 | 0.0000 |
| *Bromus catharticus* | Brome grass | monocots | Poaceae | I | 0.4407 | 1.0516 | 0.3556 | 1.1809 | 0.0668 |
| *Bromus danthoniae var. danthoniae* | Oat brome; three-awned brome | monocots | Poaceae | I | 0.0000 | 0.0699 | 0.0001 | 0.0000 | 0.0003 |
| *Bromus danthoniae var. pauciaristatus* | Oat brome; three-awned brome | monocots | Poaceae | I | 0.0000 | 0.0189 | 0.0000 | 0.0000 | 0.0000 |
| *Bromus inermis* |  | monocots | Poaceae | I | 0.0003 | 0.0090 | 0.0000 | 0.0104 | 0.0000 |
| *Bromus nervosus* |  | monocots | Poaceae | I | 0.0000 | 0.0755 | 0.0014 | 0.0000 | 0.0017 |
| *Bromus racemosus* | Bald brome, smooth brome | monocots | Poaceae | I | 0.0034 | 8.9408 | 0.7108 | 0.0053 | 0.7298 |
| *Bromus ramosus* | Hairy brome | monocots | Poaceae | I | 0.0000 | 0.0066 | 0.0163 | 0.0015 | 0.0004 |
| *Calotis lappulacea* | Yellow burr-daisy | eudicots | Asteraceae | N | 0.0019 | 0.0000 | 0.0338 | 0.0000 | 0.5185 |
| *Calystegia* spp*.* |  | eudicots | Convolvulaceae |  | 0.0000 | 0.0559 | 0.0000 | 0.0000 | 0.0000 |
| *Cardamine hirsuta* | Hairy bittercress | eudicots | Brassicaceae | I | 0.0000 | 0.0808 | 0.0002 | 0.1577 | 0.0000 |
| *Carex inversa* | Knob sedge | monocots | Cyperaceae | N | 0.0465 | 0.0000 | 0.0155 | 0.0004 | 0.0000 |
| *Cenchrus americanus* | Pearl millet | monocots | Poaceae | I | 0.0330 | 0.1049 | 0.0071 | 0.2559 | 0.3593 |
| *Centipeda minima* |  | eudicots | Asteraceae | N | 0.0000 | 0.0000 | 0.0000 | 0.0146 | 0.0000 |
| *Cerastium glomeratum* | Sticky mouse-ear chickweed; clammy chickweed | eudicots | Caryophyllaceae | I | 0.0024 | 0.0000 | 0.0001 | 0.0127 | 0.0000 |
| *Chaetopogon fasciculatus* |  | monocots | Poaceae | I | 0.0000 | 0.0018 | 0.0000 | 0.0105 | 0.0027 |
| *Chenopodium album* | Lamb's quarters; melde; goosefoot; wild spinach; fat-hen | eudicots | Amaranthaceae | I | 0.0075 | 0.0000 | 0.0010 | 0.0000 | 0.0100 |
| *Chenopodium sp. 'luteo-rufi'* |  | eudicots | Amaranthaceae |  | 0.1138 | 0.0000 | 0.2263 | 0.0002 | 0.3730 |
| *Chloris gayana* | Rhodes grass | monocots | Poaceae | I | 1.5711 | 0.0111 | 0.0001 | 0.0000 | 0.0001 |
| *Chloris pectinata* | Comb chloris | monocots | Poaceae | N | 0.0083 | 0.0000 | 0.0281 | 0.0001 | 0.0000 |
| *Chloris truncata* | Australian fingergrass; windmill-grass; Australian windmill grass; simply windmill | monocots | Poaceae | N | 0.8226 | 0.0010 | 8.0405 | 0.0083 | 0.0008 |
| *Chondrilla juncea* | Skeleton weed; gum succory; devil's grass; nakedweed | eudicots | Asteraceae | I | 0.0010 | 0.0000 | 0.0481 | 0.0000 | 0.0000 |
| *Chondrilla latifolia* |  | eudicots | Asteraceae | I | 1.2143 | 0.0001 | 3.0088 | 0.0010 | 0.0126 |
| *Cicer arietinum* |  | eudicots | Fabaceae | I | 0.0000 | 0.0212 | 0.0003 | 0.0000 | 0.0000 |
| *Cichorium intybus* |  | eudicots | Asteraceae | I | 0.0000 | 0.0000 | 0.0094 | 0.0000 | 0.0000 |
| *Cineraria geraniifolia* |  | eudicots | Asteraceae | I | 0.0273 | 0.0000 | 0.0000 | 0.0000 | 0.0000 |
| *Conyza bonariensis* |  | eudicots | Asteraceae | I | 0.0104 | 0.0011 | 0.0000 | 0.0040 | 0.0056 |
| *Corymbia ferruginea subsp. ferruginea* | Bloodwood tree | eudicots | Myrtaceae | N | 0.0120 | 0.0000 | 0.0000 | 0.0000 | 0.0000 |
| *Crassula colligata* |  | eudicots | Crassulaceae | N | 0.0188 | 0.0000 | 0.0329 | 0.0004 | 0.0000 |
| *Cynodon dactylon* | Bermuda grass; couch grass | monocots | Poaceae | N | 3.8274 | 25.8603 | 10.4329 | 0.3401 | 19.3911 |
| *Cynodon hirsutus* |  | monocots | Poaceae | I | 0.2136 | 0.0040 | 5.6584 | 0.0051 | 3.1363 |
| *Cynodon incompletus* | Blue couch grass | monocots | Poaceae | I | 0.6234 | 0.0106 | 1.3464 | 0.0066 | 6.3475 |
| *Cyperus brevifolioides* | Asian green-headed sedge | monocots | Cyperaceae | I | 0.0443 | 0.0303 | 0.0810 | 0.0058 | 0.1137 |
| *Cyperus eragrostis* | Tall flatsedge, nutgrass, tall nutgrass, umbrella sedge, chufa, Earth almond, zula nuts, edible galingale; pale galingale | monocots | Cyperaceae | I | 0.0000 | 0.0295 | 0.0001 | 0.0003 | 0.0000 |
| *Dactylis glomerata* | Cocksfoot; orchard grass | monocots | Poaceae | I | 0.0015 | 1.2901 | 0.0174 | 40.9147 | 0.0868 |
| *Dactylis glomerata subsp. hispanica* |  | monocots | Poaceae | I | 0.0000 | 0.0988 | 0.0003 | 1.3754 | 0.0032 |
| *Dichelachne sp. JS-2008* | Plumegrass | monocots | Poaceae | N | 0.0709 | 0.0173 | 0.0000 | 0.0000 | 0.0000 |
| *Dichondra repens* | Kidney weed; Mercury Bay weed; tom thumb; yilibili (Dharawal language | eudicots | Convolvulaceae | N | 1.1919 | 0.0000 | 0.0055 | 0.0359 | 0.0000 |
| *Digitaria californica var. villosissima* |  | monocots | Poaceae | I | 0.0006 | 0.0000 | 0.0000 | 0.0000 | 0.0125 |
| *Digitaria catamarcensis* |  | monocots | Poaceae | I | 0.0052 | 0.0000 | 0.0055 | 0.0000 | 0.0008 |
| *Digitaria cognata* |  | monocots | Poaceae | I | 3.9975 | 0.0009 | 0.5103 | 0.0087 | 9.5997 |
| *Digitaria ischaemum* |  | monocots | Poaceae | I | 0.0000 | 0.0000 | 0.0002 | 0.0352 | 0.0000 |
| *Digitaria setigera* |  | monocots | Poaceae | N | 1.5195 | 0.0006 | 0.0045 | 0.0851 | 0.2047 |
| *Digitaria tenuis* |  | monocots | Poaceae | I | 0.4399 | 0.0006 | 0.0646 | 0.0063 | 1.1560 |
| *Dysphania pumilio* |  | eudicots | Amaranthaceae | N | 0.2415 | 0.0000 | 0.0017 | 0.0000 | 0.0905 |
| *Echinochloa crus-galli* |  | monocots | Poaceae | I | 0.0218 | 0.0000 | 0.0000 | 0.0000 | 0.0000 |
| *Echium plantagineum* | Patterson's curse; purple viper's-bugloss | eudicots | Boraginaceae | I | 1.4381 | 0.0000 | 0.0000 | 0.1162 | 0.0026 |
| *Ectrosia schultzii* |  | monocots | Poaceae | N | 12.1620 | 0.1139 | 1.6457 | 0.0390 | 0.0171 |
| *Eleusine indica* | Crowsfoot grass | monocots | Poaceae | I | 0.0295 | 0.0000 | 0.0038 | 0.0008 | 0.0081 |
| *Eleusine tristachya* | Goose Grass, American Crowsfoot Grass, Crab Grass | monocots | Poaceae | I | 2.6868 | 0.0003 | 0.9743 | 0.0732 | 4.0712 |
| *Enneapogon asperatus* |  | monocots | Poaceae | N | 0.0000 | 0.0000 | 0.0935 | 0.0001 | 0.0000 |
| *Enteropogon ramosus* |  | monocots | Poaceae | N | 0.0000 | 0.0000 | 0.1835 | 0.0006 | 0.0000 |
| *Entolasia stricta* |  | monocots | Poaceae | N | 0.2472 | 2.0720 | 0.4743 | 5.0238 | 0.6613 |
| *Eragrostis curvula* |  | monocots | Poaceae | I | 0.0002 | 1.5059 | 0.0001 | 0.0000 | 0.0000 |
| *Eragrostis dielsii* | Mallee lovegrass | monocots | Poaceae | N | 0.0446 | 0.0021 | 0.0000 | 0.0000 | 0.0001 |
| *Eragrostis rotifer* |  | monocots | Poaceae | I | 0.1318 | 0.0019 | 0.0000 | 0.0000 | 0.0515 |
| *Eragrostis tenuifolia* | Elastic grass | monocots | Poaceae | I | 7.1134 | 0.4738 | 0.0289 | 0.0814 | 2.9431 |
| *Erigeron canadensis* |  | eudicots | Asteraceae | I | 0.2209 | 0.0000 | 0.0000 | 0.0001 | 0.0000 |
| *Eucalyptus grandis* | Flooded Gum, Rose Gum | eudicots | Myrtaceae | N | 0.4573 | 0.0045 | 0.0047 | 0.0047 | 0.0328 |
| *Eucalyptus melanoleuca* |  | eudicots | Myrtaceae | N | 0.0018 | 0.0092 | 0.0003 | 0.0000 | 0.0037 |
| *Eucalyptus neglecta* | Omeo Gum | eudicots | Myrtaceae | N | 0.0095 | 0.0000 | 0.0000 | 0.0002 | 0.0000 |
| *Eucalyptus sparsa* | Northern ranges box | eudicots | Myrtaceae | N | 0.0030 | 0.0000 | 0.0144 | 0.0002 | 0.0001 |
| *Eucalyptus thozetiana* |  | eudicots | Myrtaceae | N | 0.1468 | 0.0405 | 0.5988 | 0.0004 | 0.0133 |
| *Eucalyptus youmanii* | Youman's stringybark | eudicots | Myrtaceae | N | 0.8155 | 0.0011 | 0.0000 | 0.0001 | 0.0000 |
| *Euchiton japonicus* |  | eudicots | Asteraceae | N | 0.1370 | 0.0000 | 0.0238 | 0.0777 | 0.0000 |
| *Festuca arundinacea* | Tall fescue | monocots | Poaceae | I | 0.0000 | 0.0045 | 0.0000 | 0.0922 | 0.0001 |
| *Festuca arundinacea subsp. cirtensis* |  | monocots | Poaceae | I | 0.0000 | 0.0158 | 0.0000 | 0.0004 | 0.0000 |
| *Festuca bromoides* |  | monocots | Poaceae | I | 3.7673 | 1.9163 | 0.0001 | 0.1066 | 0.0070 |
| *Festuca myuros f. myuros* |  | monocots | Poaceae | I | 0.3432 | 0.2347 | 0.0000 | 0.0115 | 0.0011 |
| *Festuca pratensis* |  | monocots | Poaceae | I | 0.0000 | 0.1264 | 0.0000 | 0.0242 | 0.0001 |
| *Festuca rubra* | Red fescue | monocots | Poaceae | I | 0.0000 | 0.0000 | 0.0027 | 0.2166 | 0.0000 |
| *Fragaria vesca* | Woodland strawberry | eudicots | Rosaceae | I | 0.0250 | 0.0067 | 0.0000 | 0.0000 | 0.0781 |
| *Gamochaeta americana* |  | eudicots | Asteraceae | I | 0.0010 | 0.0577 | 0.0001 | 0.0205 | 0.0000 |
| *Geranium aff. homeanum RCG-2004* |  | eudicots | Geraniaceae | N | 0.2030 | 0.0266 | 0.0000 | 0.0613 | 0.0041 |
| *Glycine stenophita* |  | eudicots | Fabaceae | N | 0.1270 | 0.1758 | 2.7695 | 0.0038 | 1.0027 |
| *Glycine tabacina* |  | eudicots | Fabaceae | N | 0.0006 | 0.0000 | 0.0228 | 0.0000 | 0.0066 |
| *Gratiola officinalis* |  | eudicots | Plantaginaceae | I | 0.0096 | 0.0000 | 0.0000 | 0.0000 | 0.0000 |
| *Haloragis erecta* | Shrubby haloragis, toatoa or erect seaberry, | eudicots | Haloragaceae | I | 0.5694 | 0.0001 | 0.0001 | 0.0001 | 0.0000 |
| *Harpachne harpachnoides* |  | monocots | Poaceae | I | 0.2143 | 0.0001 | 0.0000 | 0.0009 | 0.0005 |
| *Hedypnois rhagadioloides* | Cretanweed; scaly hawkbit | eudicots | Asteraceae | I | 0.0000 | 0.0000 | 0.0214 | 0.0000 | 0.0000 |
| *Holcus annuus subsp. duriensis* |  | monocots | Poaceae | I | 0.0000 | 0.0017 | 0.0000 | 0.0195 | 0.0040 |
| *Holcus lanatus* | Yorkshire fog, tufted grass, and meadow soft grass. | monocots | Poaceae | I | 0.0438 | 4.3702 | 0.0049 | 12.7572 | 2.3170 |
| *Hordeum murinum subsp. glaucum* |  | monocots | Poaceae | I | 0.0000 | 0.0001 | 1.5450 | 0.0063 | 0.0000 |
| *Hymenachne grumosa* |  | monocots | Poaceae | I | 0.0328 | 0.2751 | 8.9438 | 0.2434 | 0.0418 |
| *Hypericum perforatum* | St John's Wort | eudicots | Hypericaceae | I | 0.2798 | 0.0000 | 0.0000 | 0.0000 | 0.0000 |
| *Hypochaeris* spp*.* |  | eudicots | Asteraceae |  | 4.1132 | 0.3138 | 0.0098 | 2.6870 | 0.0237 |
| *Hypochaeris glabra* | Smooth cats-ear; annual flatweed. | eudicots | Asteraceae | I | 0.1060 | 0.0014 | 0.0000 | 0.0041 | 0.0000 |
| *Isolepis prolifera* |  | monocots | Cyperaceae | I | 0.0000 | 0.0105 | 0.0000 | 0.0147 | 0.0000 |
| *Lachnagrostis littoralis subsp. salaria* |  | monocots | Poaceae | I | 0.0172 | 0.0000 | 0.0000 | 0.0000 | 0.0000 |
| *Lenwebbia lasioclada* | Velvet myrtle | eudicots | Myrtaceae | N | 0.0000 | 0.0000 | 0.0000 | 0.0537 | 0.0001 |
| *Leontodon maroccanus* |  | eudicots | Asteraceae | I | 0.0083 | 0.0003 | 0.0000 | 0.0003 | 0.0000 |
| *Leontodon saxatilis* | Lesser hawkbit, rough hawkbit, and hairy hawkbit. | eudicots | Asteraceae | I | 1.0490 | 0.0372 | 0.0000 | 0.0128 | 0.0011 |
| *Leontodon saxatilis subsp. saxatilis* |  | eudicots | Asteraceae | I | 0.0069 | 0.0003 | 0.0000 | 0.0007 | 0.0000 |
| *Lolium canariense* |  | monocots | Poaceae | I | 0.0000 | 0.0028 | 0.0001 | 0.0345 | 0.0000 |
| *Lolium perenne* | Perennial ryegrass | monocots | Poaceae | I | 0.0151 | 0.5038 | 1.4370 | 10.0480 | 0.1249 |
| *Lolium persicum* | Persian ryegrass | monocots | Poaceae | I | 0.0000 | 0.0334 | 0.2079 | 0.2688 | 0.0000 |
| *Lolium rigidum* | Wimmera ryegrass; annual ryegrass | monocots | Poaceae | I | 0.0000 | 0.0176 | 0.0000 | 0.0001 | 0.0000 |
| *Lotus pedunculatus* | Big trefoil, greater bird's-foot-trefoil or marsh bird's-foot trefoil | eudicots | Fabaceae | I | 0.0000 | 0.5782 | 0.0000 | 0.0000 | 0.0000 |
| *Lotus subbiflorus* | Hairy bird's-foot trefoil | eudicots | Fabaceae | I | 0.0000 | 0.0000 | 0.0000 | 0.0403 | 0.0000 |
| *Ludwigia peploides* | Floating primrose-willow; creeping water primrose | eudicots | Onagraceae | N | 0.0001 | 0.7135 | 0.0000 | 0.0000 | 0.0000 |
| *Malva verticillata* | Chinese mallow or cluster mallow | eudicots | Malvaceae | I | 0.0001 | 0.0000 | 3.9479 | 0.2170 | 0.0002 |
| *Medicago falcata* |  | eudicots | Fabaceae | I | 1.3664 | 0.0000 | 0.0017 | 0.0000 | 0.0000 |
| *Modiola caroliniana* | Bristly-fruited mallow, Carolina bristlemallow, babosilla, and redflower mallow | eudicots | Malvaceae | I | 0.3125 | 0.0022 | 0.0321 | 0.0077 | 0.0646 |
| *Myriophyllum sp. Les 542* |  | eudicots | Haloragaceae | N | 0.2300 | 0.0001 | 0.0002 | 0.0000 | 0.0000 |
| *Nasturtium officinale* | Watercress; yellowcress | eudicots | Brassicaceae | I | 0.0000 | 0.0494 | 0.0000 | 0.0000 | 0.0000 |
| *Oenothera lindheimeri* |  | eudicots | Onagraceae | I | 0.0000 | 0.0000 | 0.0002 | 1.9419 | 0.0000 |
| *Oenothera versicolor* |  | eudicots | Onagraceae | I | 0.0328 | 0.0000 | 0.0000 | 0.0007 | 0.0000 |
| *Oplismenus undulatifolius* | Wavyleaf basketgrass | monocots | Poaceae | I | 0.0000 | 1.3287 | 0.0001 | 0.6841 | 0.6613 |
| *Ornithopus pinnatus* | Orange birdsfoot | eudicots | Fabaceae | I | 0.0001 | 0.0000 | 0.0013 | 0.6981 | 0.0001 |
| *Oxalis corniculata* |  | eudicots | Oxalidaceae | I | 0.2328 | 0.0226 | 0.0510 | 0.0172 | 0.0083 |
| *Panicum pauciflorum* |  | monocots | Poaceae | N | 10.0566 | 0.0014 | 0.3730 | 0.0204 | 0.2401 |
| *Panicum queenslandicum* |  | monocots | Poaceae | N | 0.0546 | 0.0000 | 0.7279 | 0.0008 | 0.6907 |
| *Panicum repens* |  | monocots | Poaceae | N | 0.6477 | 0.0001 | 0.0007 | 0.6636 | 0.0001 |
| *Panicum schinzii* |  | monocots | Poaceae | I | 0.0000 | 0.0000 | 0.0000 | 0.0274 | 0.0314 |
| *Paronychia baldwinii* |  | eudicots | Caryophyllaceae | I | 1.7523 | 0.0000 | 0.2546 | 0.0031 | 0.0001 |
| *Paspalidium constrictum* | Knottybutt grass; box grass; knottybutt paspalidium | monocots | Poaceae | N | 0.0028 | 0.0050 | 16.1682 | 0.0241 | 0.0020 |
| *Paspalum dilatatum* | Dallis grass | monocots | Poaceae | I | 11.6264 | 26.8717 | 8.6974 | 9.8614 | 37.9901 |
| *Paspalum distichum* | Knotgrass; water finger-grass; couch paspalum; eternity grass; gingergrass; Thompson grass | monocots | Poaceae | I | 0.0024 | 0.0115 | 0.2959 | 0.0006 | 0.0105 |
| *Paspalum distichum var. indutum* |  | monocots | Poaceae | I | 0.0539 | 0.1709 | 1.4252 | 0.0017 | 0.1336 |
| *Perotis rara* |  | monocots | Poaceae | N | 0.0001 | 0.0001 | 0.9088 | 0.0001 | 0.0000 |
| *Petrorhagia nanteuilii* | Childing pink; productive carnation; proliferous pink; wild carnation | eudicots | Caryophyllaceae | I | 0.0521 | 0.0000 | 0.0000 | 0.0000 | 0.0000 |
| *Phalaris angusta* | Timothy canarygrass; narrow canarygrass | monocots | Poaceae | I | 0.1223 | 0.0017 | 0.0000 | 0.0000 | 0.0002 |
| *Phalaris aquatica* | Bulbous canary-grass; harding grass | monocots | Poaceae | I | 2.9367 | 0.0257 | 0.0000 | 0.0038 | 0.0287 |
| *Phalaris arundinacea* | Reed canary grass | monocots | Poaceae | I | 0.0093 | 0.0675 | 0.0000 | 0.0000 | 0.0000 |
| *Phalaris arundinacea var. arundinacea* |  | monocots | Poaceae | I | 0.0069 | 2.5023 | 0.0004 | 0.0000 | 0.0000 |
| *Phalaris peruviana* |  | monocots | Poaceae | I | 0.0046 | 0.0092 | 0.0000 | 0.0000 | 0.0000 |
| *Pinus contorta* | Lodgepole pine; shore pine; twisted pine; contorta pine | seed plants | Pinaceae | I | 0.0762 | 0.0112 | 0.0000 | 0.0525 | 0.0000 |
| *Pittosporum undulatum* | Sweet pittosporum; native daphne; Australian cheesewood, Victorian box; mock orange | eudicots | Pittosporaceae | N | 0.0000 | 0.3541 | 0.0000 | 0.0138 | 0.0000 |
| *Plantago lanceolata* | Ribwort plantain; narrowleaf plantain; English plantain; ribleaf; lamb's tongue; buckhorn | eudicots | Plantaginaceae | I | 0.2549 | 0.9664 | 0.0004 | 0.0814 | 1.7119 |
| *Poa affinis* |  | monocots | Poaceae | N | 0.1559 | 0.1013 | 0.0000 | 0.1449 | 0.0033 |
| *Poa annua* | Annual meadow grass | monocots | Poaceae | I | 0.0000 | 0.0000 | 0.0002 | 0.4738 | 0.0001 |
| *Poa bulbosa* | Bulbous bluegrass; bulbous meadow-grass | monocots | Poaceae | I | 0.0000 | 0.0000 | 0.0000 | 0.0153 | 0.0000 |
| *Poa chaixii* | Broad-leaved meadow-grass; broadleaf bluegrass | monocots | Poaceae | I | 0.0008 | 0.0091 | 0.0000 | 0.0000 | 0.0000 |
| *Poa iconia var. pelasgis* |  | monocots | Poaceae | I | 0.0083 | 0.0733 | 0.0000 | 0.0268 | 0.0008 |
| *Poa infirma* | Early meadow-grass; weak bluegrass | monocots | Poaceae | I | 0.0488 | 0.0000 | 0.0002 | 0.1218 | 0.0001 |
| *Poa orthoclada* |  | monocots | Poaceae | N | 0.0050 | 0.0071 | 0.0000 | 0.0000 | 0.0016 |
| *Poa pratensis* | Kentucky bluegrass | monocots | Poaceae | I | 0.0000 | 0.0080 | 0.0000 | 0.0061 | 0.0000 |
| *Poa trivialis* | Rough meadow grass | monocots | Poaceae | I | 0.0000 | 0.0992 | 0.0000 | 0.0460 | 0.0230 |
| *Polycarpon tetraphyllum* | Four-leaved allseed | eudicots | Caryophyllaceae | I | 0.0760 | 0.0000 | 0.0000 | 0.0000 | 0.0000 |
| *Pomax umbellata* |  | eudicots | Rubiaceae | N | 2.5922 | 0.0002 | 0.0732 | 0.0000 | 0.0005 |
| *Prunus sibirica* | Siberian apricot | eudicots | Rosaceae | I | 0.1514 | 0.0273 | 0.0000 | 0.0000 | 0.0000 |
| *Pseudognaphalium affine* |  | eudicots | Asteraceae | I | 0.0690 | 0.0007 | 0.0000 | 0.0000 | 0.0000 |
| *Rosa banksiae* | Lady Bank's rose; Bank's rose | eudicots | Rosaceae | I | 0.0000 | 0.0000 | 0.0000 | 0.2121 | 0.0000 |
| *Rosa x damascena;Rosa moschata;Rosa chinensis;Rosa dumalis* |  | eudicots | Rosaceae | I | 0.0000 | 0.0000 | 0.0000 | 0.0184 | 0.0000 |
| *Rubus sp. MS-2014k* |  | eudicots | Rosaceae | I | 0.0000 | 3.1844 | 0.0000 | 0.0562 | 0.0120 |
| *Rubus swinhoei* | [Swinhoe's raspberry; wood berry; Keelung rubus; Jingbai rubus; Libai rubus](https://en.wikipedia.org/wiki/Rubus_swinhoei) | eudicots | Rosaceae | I | 0.0000 | 0.0131 | 0.0000 | 0.0000 | 0.0002 |
| *Rytidosperma auriculatum* | Lobed wallaby grass | monocots | Poaceae | N | 0.0465 | 0.0000 | 0.0000 | 0.0000 | 0.0000 |
| *Rytidosperma caespitosum* | Common wallaby-grass; ringed wallaby-grass; white-top, | monocots | Poaceae | N | 0.0689 | 0.0000 | 0.0000 | 0.0000 | 0.0050 |
| *Rytidosperma clelandii* |  | monocots | Poaceae | N | 0.0281 | 0.0000 | 0.0061 | 0.0000 | 0.0000 |
| *Rytidosperma gracile* | Dainty bristle grass | monocots | Poaceae | N | 0.0467 | 0.0001 | 0.0140 | 0.0000 | 0.0000 |
| *Rytidosperma longifolium* |  | monocots | Poaceae | N | 0.1746 | 0.0001 | 0.0000 | 0.0001 | 0.0000 |
| *Rytidosperma merum* |  | monocots | Poaceae | I | 0.0110 | 0.0000 | 0.0000 | 0.0000 | 0.0000 |
| *Rytidosperma penicillatum* | Slender wallaby grass | monocots | Poaceae | N | 0.1424 | 0.0000 | 0.0018 | 0.0000 | 0.0000 |
| *Rytidosperma pictum* |  | monocots | Poaceae | I | 0.0089 | 0.0003 | 0.0070 | 0.0000 | 0.0009 |
| *Rytidosperma pulchrum* |  | monocots | Poaceae | I | 0.0359 | 0.0002 | 0.0000 | 0.0000 | 0.0016 |
| *Rytidosperma racemosum* | Wallaby grass | monocots | Poaceae | N | 5.7701 | 0.0088 | 0.0414 | 0.0034 | 0.0531 |
| *Rytidosperma sp. Humphreys 104* |  | monocots | Poaceae |  | 0.0656 | 0.0106 | 0.0780 | 0.0000 | 0.0007 |
| *Rytidosperma telmaticum* | Tarn bristle grass | monocots | Poaceae | I | 0.0134 | 0.0000 | 0.0000 | 0.0000 | 0.0000 |
| *Setaria parviflora* | Marsh bristlegrass, knotroot bristle-grass, bristly foxtail and yellow bristlegrass | monocots | Poaceae | I | 0.0852 | 0.2659 | 0.0083 | 0.0008 | 0.0301 |
| *Setaria pumila* | Yellow foxtail, yellow bristle-grass, pigeon grass, and cattail grass | monocots | Poaceae | I | 0.9407 | 0.1549 | 0.1296 | 0.0152 | 2.2603 |
| *Silene gallica* | Catchfly, small-flowered catchfly, and windmill pink. | eudicots | Caryophyllaceae | I | 0.0000 | 0.0000 | 0.0000 | 0.0919 | 0.0001 |
| *Sinapis arvensis* | Charlock mustard, field mustard, wild mustard, charlock | eudicots | Brassicaceae | I | 0.0000 | 0.0000 | 0.0156 | 0.0000 | 0.0000 |
| *Solenogyne dominii* |  | eudicots | Asteraceae | N | 0.0305 | 0.0000 | 0.0000 | 0.0000 | 0.0000 |
| *Soliva sessilis* | Field burrweed, Onehunga-weed, lawn burrweed, lawnweed, jo-jo weed; common soliva | eudicots | Asteraceae | I | 0.0110 | 0.0000 | 0.0000 | 0.0000 | 0.0000 |
| *Sonchus oleraceus* | Common sowthistle, sow thistle, smooth sow thistle, annual sow thistle, hare's colwort, hare's thistle, milky tassel, milk thistle; soft thistle | eudicots | Asteraceae | I | 0.0023 | 0.0145 | 0.0000 | 0.0000 | 0.0000 |
| *Sporobolus fertilis* | Giant parramatta grass | monocots | Poaceae | I | 0.0035 | 0.0002 | 0.0095 | 0.0001 | 0.0001 |
| *Stellaria longifolia* | Long leaf starwort | eudicots | Caryophyllaceae | I | 1.2995 | 0.0101 | 0.0000 | 0.0000 | 0.0000 |
| *Stellaria pallida* | Lesser chickweed | eudicots | Caryophyllaceae | I | 0.0924 | 0.0639 | 0.0001 | 0.0085 | 0.0001 |
| *Symphyotrichum subulatum* | Saltmarsh aster | eudicots | Asteraceae | I | 0.4264 | 0.0002 | 1.0591 | 0.0066 | 0.0000 |
| *Symphyotrichum subulatum var. squamatum* |  | eudicots | Asteraceae | I | 0.0006 | 0.0000 | 0.0185 | 0.0000 | 0.0000 |
| *Tagetes minuta* | Southern cone marigold | eudicots | Asteraceae | I | 0.0000 | 0.0232 | 0.0000 | 0.0000 | 0.0000 |
| *Taraxacum officinale* | Dandelion | eudicots | Asteraceae | I | 0.0001 | 0.0598 | 0.0000 | 0.1370 | 0.0013 |
| *Themeda triandra* | Kangaroo grass | monocots | Poaceae | I | 0.0000 | 0.0021 | 0.0000 | 0.0000 | 0.6476 |
| *Thinopyrum elongatum* | Tall wheatgrass | monocots | Poaceae | I | 0.5485 | 0.0000 | 0.0000 | 0.0000 | 0.0048 |
| *Tragus andicola* |  | monocots | Poaceae | I | 0.0000 | 0.0000 | 0.2492 | 0.0001 | 0.0000 |
| *Tribulus terrestris* |  | eudicots | Zygophyllaceae | I | 0.0000 | 0.0000 | 0.0324 | 0.0000 | 0.0000 |
| *Trifolium arvense* | Hare's-foot clover, rabbitfoot clover, stone clover or oldfield clover, | eudicots | Fabaceae | I | 0.0800 | 0.0000 | 0.0000 | 0.0000 | 0.0001 |
| *Trifolium aureum* | large hop trefoil, large trefoil, large hop clover, golden clover or hop clover | eudicots | Fabaceae | I | 0.0251 | 0.0000 | 0.0000 | 0.0008 | 0.0000 |
| *Trifolium* spp*.* | Clovers | eudicots | Fabaceae |  | 0.0390 | 0.0166 | 0.0022 | 1.8046 | 0.0004 |
| *Trifolium glomeratum* | Clustered clover and bush clover | eudicots | Fabaceae | I | 0.6726 | 0.0001 | 0.0001 | 0.0042 | 0.0000 |
| *Trifolium nigrescens subsp. petrisavii* |  | eudicots | Fabaceae | I | 0.2537 | 0.0000 | 0.0001 | 0.0106 | 0.0000 |
| *Trifolium repens* | White clover | eudicots | Fabaceae | I | 0.0001 | 0.0008 | 0.0003 | 0.1253 | 0.0000 |
| *Triodia scariosa* | Porcupine grass or spinifex | monocots | Poaceae | N | 0.0000 | 0.0000 | 0.0249 | 0.0000 | 0.0000 |
| *Triticum turgidum subsp. durum* | Durum wheat | monocots | Poaceae | I | 0.0168 | 0.0000 | 0.0000 | 0.0000 | 0.0000 |
| Unassigned spp. |  |  |  |  | 2.1140 | 0.5696 | 1.7113 | 0.5112 | 0.2598 |
| *Urochloa trichopus* | Bushveld signal grass | monocots | Poaceae | I | 0.0004 | 0.0003 | 3.3378 | 0.0030 | 0.0014 |
| *Veronica anagallis-aquatica* | Water speedwell; blue water-speedwell; brook pimpernel; sessile water-speedwell | eudicots | Plantaginaceae | I | 0.0000 | 0.0801 | 0.0001 | 0.0000 | 0.0000 |
| *Veronica arvensis* | Wall speedwell; corn speedwell; common speedwell; rock speedwell; field speedwell | eudicots | Plantaginaceae | I | 0.0079 | 0.0000 | 0.0000 | 0.0001 | 0.0000 |
| *Vicia nigricans subsp. gigantea* | Giant vetch | eudicots | Fabaceae | I | 0.0485 | 0.0327 | 0.0000 | 0.0007 | 0.0000 |

I = Introduced; N = native

Table S2. Frequency of occurrence of plant species at each study site in the scats of bare-nosed wombats

| Scientific name | Badger Ground | Coolagolite | Merriwa | Robertson | Wolgan Valley |
| --- | --- | --- | --- | --- | --- |
| *Acaena caesiglauca* | 47.4 | 0.0 | 0.0 | 0.0 | 0.0 |
| *Agrostis castellana* | 0.0 | 26.3 | 0.0 | 65.0 | 0.0 |
| *Agrostis mertensii* | 5.3 | 68.4 | 0.0 | 65.0 | 10.0 |
| *Anagallis arvensis* | 0.0 | 5.3 | 0.0 | 0.0 | 0.0 |
| *Angophora costata* | 26.3 | 0.0 | 0.0 | 0.0 | 0.0 |
| *Anthosachne aprica* | 36.8 | 0.0 | 0.0 | 0.0 | 25.0 |
| *Anthoxanthum alpinum* | 0.0 | 26.3 | 0.0 | 85.0 | 0.0 |
| *Anthoxanthum aristatum* | 0.0 | 68.4 | 28.6 | 100.0 | 10.0 |
| *Anthoxanthum odoratum* | 0.0 | 47.4 | 0.0 | 95.0 | 0.0 |
| *Anthoxanthum ovatum* | 0.0 | 10.5 | 0.0 | 85.0 | 0.0 |
| *Anthoxanthum sp. PT-2016* | 10.5 | 73.7 | 28.6 | 100.0 | 15.0 |
| *Arrhenatherum elatius* | 0.0 | 100.0 | 28.6 | 35.0 | 15.0 |
| *Austrostipa rudis* | 21.1 | 26.3 | 85.7 | 15.0 | 50.0 |
| *Austrostipa rudis subsp. nervosa* | 10.5 | 10.5 | 57.1 | 5.0 | 25.0 |
| *Austrostipa scabra* | 63.2 | 5.3 | 100.0 | 30.0 | 35.0 |
| *Austrostipa scabra subsp. Falcata* | 31.6 | 10.5 | 64.3 | 0.0 | 5.0 |
| *Austrostipa trichophylla* | 10.5 | 0.0 | 57.1 | 0.0 | 5.0 |
| *Austrostipa verticillata* | 5.3 | 0.0 | 85.7 | 10.0 | 25.0 |
| *Avena sativa* | 36.8 | 21.1 | 7.1 | 20.0 | 0.0 |
| *Axonopus argentinus* | 0.0 | 5.3 | 0.0 | 5.0 | 0.0 |
| *Axonopus polystachyus* | 10.5 | 100.0 | 42.9 | 30.0 | 10.0 |
| *Boerhavia erecta* | 0.0 | 0.0 | 57.1 | 0.0 | 0.0 |
| *Bothriochloa ischaemum* | 42.1 | 0.0 | 35.7 | 0.0 | 60.0 |
| *Brachiaria subquadripara* | 5.3 | 0.0 | 78.6 | 5.0 | 45.0 |
| *Briza minor* | 21.1 | 0.0 | 0.0 | 5.0 | 0.0 |
| *Bromus catharticus* | 73.7 | 78.9 | 64.3 | 80.0 | 35.0 |
| *Bromus danthoniae var. danthoniae* | 0.0 | 31.6 | 7.1 | 0.0 | 5.0 |
| *Bromus danthoniae var. pauciaristatus* | 0.0 | 15.8 | 0.0 | 0.0 | 0.0 |
| *Bromus inermis* | 5.3 | 26.3 | 0.0 | 15.0 | 0.0 |
| *Bromus nervosus* | 0.0 | 31.6 | 14.3 | 0.0 | 10.0 |
| *Bromus racemosus* | 10.5 | 100.0 | 57.1 | 30.0 | 45.0 |
| *Bromus ramosus* | 0.0 | 10.5 | 14.3 | 5.0 | 10.0 |
| *Calotis lappulacea* | 5.3 | 0.0 | 28.6 | 0.0 | 15.0 |
| *Calystegia* spp. | 0.0 | 5.3 | 0.0 | 0.0 | 0.0 |
| *Cardamine hirsuta* | 0.0 | 26.3 | 14.3 | 30.0 | 0.0 |
| *Carex inversa* | 73.7 | 0.0 | 28.6 | 10.0 | 0.0 |
| *Cenchrus americanus* | 47.4 | 68.4 | 35.7 | 100.0 | 80.0 |
| *Centipeda minima* | 0.0 | 0.0 | 0.0 | 5.0 | 0.0 |
| *Cerastium glomeratum* | 5.3 | 0.0 | 7.1 | 10.0 | 0.0 |
| *Chaetopogon fasciculatus* | 0.0 | 21.1 | 0.0 | 45.0 | 10.0 |
| *Chenopodium album* | 10.5 | 0.0 | 21.4 | 0.0 | 15.0 |
| *Chenopodium sp. 'luteo-rufi'* | 21.1 | 0.0 | 57.1 | 10.0 | 15.0 |
| *Chloris gayana* | 89.5 | 26.3 | 7.1 | 0.0 | 5.0 |
| *Chloris pectinata* | 42.1 | 0.0 | 64.3 | 5.0 | 0.0 |
| *Chloris truncata* | 89.5 | 42.1 | 100.0 | 30.0 | 15.0 |
| *Chondrilla juncea* | 15.8 | 0.0 | 28.6 | 0.0 | 0.0 |
| *Chondrilla latifolia* | 68.4 | 5.3 | 92.9 | 15.0 | 25.0 |
| *Cicer arietinum* | 0.0 | 26.3 | 7.1 | 0.0 | 0.0 |
| *Cichorium intybus* | 0.0 | 0.0 | 7.1 | 0.0 | 0.0 |
| *Cineraria geraniifolia* | 26.3 | 0.0 | 0.0 | 0.0 | 0.0 |
| *Conyza bonariensis* | 15.8 | 5.3 | 0.0 | 5.0 | 5.0 |
| *Corymbia ferruginea subsp. ferruginea* | 5.3 | 0.0 | 0.0 | 0.0 | 0.0 |
| *Crassula colligata* | 15.8 | 0.0 | 7.1 | 5.0 | 0.0 |
| *Cynodon dactylon* | 100.0 | 100.0 | 100.0 | 90.0 | 95.0 |
| *Cynodon hirsutus* | 47.4 | 47.4 | 100.0 | 65.0 | 85.0 |
| *Cynodon incompletus* | 26.3 | 84.2 | 100.0 | 65.0 | 85.0 |
| *Cyperus brevifolioides* | 42.1 | 52.6 | 42.9 | 10.0 | 75.0 |
| *Cyperus eragrostis* | 0.0 | 47.4 | 7.1 | 5.0 | 0.0 |
| *Dactylis glomerata* | 63.2 | 94.7 | 71.4 | 100.0 | 55.0 |
| *Dactylis glomerata subsp. hispanica* | 0.0 | 57.9 | 28.6 | 100.0 | 10.0 |
| *Dichelachne sp. JS-2008* | 42.1 | 10.5 | 0.0 | 0.0 | 0.0 |
| *Dichondra repens* | 73.7 | 0.0 | 7.1 | 15.0 | 0.0 |
| *Digitaria californica var. villosissima* | 10.5 | 0.0 | 0.0 | 0.0 | 50.0 |
| *Digitaria catamarcensis* | 47.4 | 0.0 | 57.1 | 0.0 | 15.0 |
| *Digitaria cognata* | 100.0 | 36.8 | 92.9 | 80.0 | 90.0 |
| *Digitaria ischaemum* | 0.0 | 0.0 | 14.3 | 10.0 | 0.0 |
| *Digitaria setigera* | 84.2 | 15.8 | 42.9 | 20.0 | 50.0 |
| *Digitaria tenuis* | 68.4 | 26.3 | 78.6 | 30.0 | 90.0 |
| *Dysphania pumilio* | 15.8 | 0.0 | 14.3 | 0.0 | 5.0 |
| *Echinochloa crus-galli* | 15.8 | 0.0 | 0.0 | 0.0 | 0.0 |
| *Echium plantagineum* | 63.2 | 0.0 | 0.0 | 5.0 | 20.0 |
| *Ectrosia schultzii* | 100.0 | 52.6 | 92.9 | 40.0 | 45.0 |
| *Eleusine indica* | 57.9 | 0.0 | 42.9 | 10.0 | 40.0 |
| *Eleusine tristachya* | 78.9 | 10.5 | 64.3 | 60.0 | 85.0 |
| *Enneapogon asperatus* | 0.0 | 0.0 | 42.9 | 5.0 | 0.0 |
| *Enteropogon ramosus* | 0.0 | 0.0 | 50.0 | 15.0 | 0.0 |
| *Entolasia stricta* | 73.7 | 100.0 | 64.3 | 95.0 | 40.0 |
| *Eragrostis curvula* | 15.8 | 63.2 | 7.1 | 0.0 | 0.0 |
| *Eragrostis dielsii* | 68.4 | 5.3 | 0.0 | 0.0 | 5.0 |
| *Eragrostis rotifer* | 57.9 | 15.8 | 0.0 | 0.0 | 25.0 |
| *Eragrostis tenuifolia* | 100.0 | 52.6 | 42.9 | 40.0 | 95.0 |
| *Erigeron canadensis* | 57.9 | 0.0 | 0.0 | 5.0 | 0.0 |
| *Eucalyptus grandis* | 63.2 | 5.3 | 28.6 | 5.0 | 25.0 |
| *Eucalyptus melanoleuca* | 10.5 | 10.5 | 7.1 | 0.0 | 10.0 |
| *Eucalyptus neglecta* | 10.5 | 0.0 | 0.0 | 5.0 | 0.0 |
| *Eucalyptus sparsa* | 21.1 | 0.0 | 28.6 | 5.0 | 5.0 |
| *Eucalyptus thozetiana* | 52.6 | 10.5 | 50.0 | 15.0 | 15.0 |
| *Eucalyptus youmanii* | 57.9 | 5.3 | 0.0 | 5.0 | 0.0 |
| *Euchiton japonicus* | 36.8 | 0.0 | 7.1 | 15.0 | 0.0 |
| *Festuca arundinacea* | 0.0 | 47.4 | 0.0 | 95.0 | 5.0 |
| *Festuca arundinacea subsp. cirtensis* | 0.0 | 15.8 | 0.0 | 5.0 | 0.0 |
| *Festuca bromoides* | 89.5 | 89.5 | 7.1 | 70.0 | 30.0 |
| *Festuca myuros f. myuros* | 42.1 | 26.3 | 0.0 | 15.0 | 15.0 |
| *Festuca pratensis* | 0.0 | 47.4 | 0.0 | 45.0 | 5.0 |
| *Festuca rubra* | 0.0 | 0.0 | 7.1 | 20.0 | 0.0 |
| *Fragaria vesca* | 36.8 | 21.1 | 0.0 | 0.0 | 5.0 |
| *Gamochaeta americana* | 5.3 | 31.6 | 7.1 | 15.0 | 0.0 |
| *Geranium aff. homeanum RCG-2004* | 36.8 | 15.8 | 0.0 | 35.0 | 5.0 |
| *Glycine stenophita* | 57.9 | 36.8 | 92.9 | 25.0 | 45.0 |
| *Glycine tabacina* | 5.3 | 0.0 | 28.6 | 0.0 | 20.0 |
| *Gratiola officinalis* | 5.3 | 0.0 | 0.0 | 0.0 | 0.0 |
| *Haloragis erecta* | 57.9 | 5.3 | 7.1 | 5.0 | 0.0 |
| *Harpachne harpachnoides* | 78.9 | 5.3 | 0.0 | 5.0 | 15.0 |
| *Hedypnois rhagadioloides* | 0.0 | 0.0 | 7.1 | 0.0 | 0.0 |
| *Holcus annuus subsp. duriensis* | 0.0 | 15.8 | 0.0 | 85.0 | 5.0 |
| *Holcus lanatus* | 26.3 | 100.0 | 57.1 | 100.0 | 50.0 |
| *Hordeum murinum subsp. glaucum* | 0.0 | 5.3 | 78.6 | 15.0 | 0.0 |
| *Hymenachne grumosa* | 36.8 | 100.0 | 100.0 | 80.0 | 55.0 |
| *Hypericum perforatum* | 47.4 | 0.0 | 0.0 | 0.0 | 0.0 |
| *Hypochaeris* spp. | 100.0 | 73.7 | 57.1 | 85.0 | 50.0 |
| *Hypochaeris glabra* | 47.4 | 10.5 | 0.0 | 15.0 | 0.0 |
| *Isolepis prolifera* | 0.0 | 10.5 | 0.0 | 5.0 | 0.0 |
| *Lachnagrostis littoralis subsp. salaria* | 21.1 | 0.0 | 0.0 | 0.0 | 0.0 |
| *Lenwebbia lasioclada* | 0.0 | 0.0 | 0.0 | 20.0 | 5.0 |
| *Leontodon maroccanus* | 42.1 | 5.3 | 0.0 | 5.0 | 0.0 |
| *Leontodon saxatilis* | 73.7 | 36.8 | 0.0 | 5.0 | 10.0 |
| *Leontodon saxatilis subsp. saxatilis* | 36.8 | 5.3 | 0.0 | 5.0 | 0.0 |
| *Lolium canariense* | 0.0 | 31.6 | 7.1 | 50.0 | 0.0 |
| *Lolium perenne* | 26.3 | 73.7 | 78.6 | 100.0 | 40.0 |
| *Lolium persicum* | 0.0 | 52.6 | 28.6 | 65.0 | 0.0 |
| *Lolium rigidum* | 0.0 | 10.5 | 0.0 | 5.0 | 0.0 |
| *Lotus pedunculatus* | 0.0 | 42.1 | 0.0 | 0.0 | 0.0 |
| *Lotus subbiflorus* | 0.0 | 0.0 | 0.0 | 15.0 | 0.0 |
| *Ludwigia peploides* | 5.3 | 52.6 | 0.0 | 0.0 | 0.0 |
| *Malva verticillata* | 5.3 | 0.0 | 85.7 | 30.0 | 5.0 |
| *Medicago falcata* | 94.7 | 0.0 | 14.3 | 0.0 | 0.0 |
| *Modiola caroliniana* | 63.2 | 5.3 | 7.1 | 10.0 | 5.0 |
| *Myriophyllum sp. Les 542* | 36.8 | 5.3 | 14.3 | 0.0 | 0.0 |
| *Nasturtium officinale* | 0.0 | 15.8 | 0.0 | 0.0 | 0.0 |
| *Oenothera lindheimeri* | 0.0 | 0.0 | 7.1 | 45.0 | 0.0 |
| *Oenothera versicolor* | 26.3 | 0.0 | 0.0 | 10.0 | 0.0 |
| *Oplismenus undulatifolius* | 0.0 | 68.4 | 7.1 | 70.0 | 10.0 |
| *Ornithopus pinnatus* | 5.3 | 0.0 | 42.9 | 15.0 | 5.0 |
| *Oxalis corniculata* | 68.4 | 31.6 | 35.7 | 15.0 | 25.0 |
| *Panicum pauciflorum* | 94.7 | 52.6 | 100.0 | 40.0 | 60.0 |
| *Panicum queenslandicum* | 68.4 | 0.0 | 100.0 | 15.0 | 30.0 |
| *Panicum repens* | 63.2 | 5.3 | 21.4 | 15.0 | 5.0 |
| *Panicum schinzii* | 0.0 | 0.0 | 0.0 | 5.0 | 15.0 |
| *Paronychia baldwinii* | 94.7 | 0.0 | 28.6 | 5.0 | 5.0 |
| *Paspalidium constrictum* | 42.1 | 94.7 | 100.0 | 80.0 | 40.0 |
| *Paspalum dilatatum* | 100.0 | 100.0 | 100.0 | 100.0 | 100.0 |
| *Paspalum distichum* | 15.8 | 15.8 | 78.6 | 20.0 | 10.0 |
| *Paspalum distichum var. indutum* | 63.2 | 52.6 | 92.9 | 45.0 | 70.0 |
| *Perotis rara* | 5.3 | 5.3 | 42.9 | 5.0 | 0.0 |
| *Petrorhagia nanteuilii* | 5.3 | 0.0 | 0.0 | 0.0 | 0.0 |
| *Phalaris angusta* | 36.8 | 21.1 | 0.0 | 0.0 | 10.0 |
| *Phalaris aquatica* | 100.0 | 21.1 | 0.0 | 5.0 | 30.0 |
| *Phalaris arundinacea* | 5.3 | 26.3 | 0.0 | 0.0 | 0.0 |
| *Phalaris arundinacea var. arundinacea* | 15.8 | 89.5 | 7.1 | 0.0 | 0.0 |
| *Phalaris peruviana* | 5.3 | 10.5 | 0.0 | 0.0 | 0.0 |
| *Pinus contorta* | 52.6 | 31.6 | 0.0 | 30.0 | 0.0 |
| *Pittosporum undulatum* | 0.0 | 36.8 | 0.0 | 15.0 | 0.0 |
| *Plantago lanceolata* | 42.1 | 78.9 | 21.4 | 60.0 | 45.0 |
| *Poa affinis* | 36.8 | 42.1 | 0.0 | 65.0 | 10.0 |
| *Poa annua* | 0.0 | 0.0 | 14.3 | 60.0 | 5.0 |
| *Poa bulbosa* | 0.0 | 0.0 | 0.0 | 10.0 | 0.0 |
| *Poa chaixii* | 5.3 | 26.3 | 0.0 | 0.0 | 0.0 |
| *Poa iconia var. pelasgis* | 31.6 | 47.4 | 0.0 | 45.0 | 10.0 |
| *Poa infirma* | 10.5 | 0.0 | 21.4 | 45.0 | 5.0 |
| *Poa orthoclada* | 21.1 | 26.3 | 0.0 | 0.0 | 10.0 |
| *Poa pratensis* | 0.0 | 26.3 | 0.0 | 40.0 | 0.0 |
| *Poa trivialis* | 0.0 | 52.6 | 0.0 | 30.0 | 10.0 |
| *Polycarpon tetraphyllum* | 26.3 | 0.0 | 0.0 | 0.0 | 0.0 |
| *Pomax umbellata* | 47.4 | 5.3 | 28.6 | 0.0 | 10.0 |
| *Prunus sibirica* | 31.6 | 21.1 | 0.0 | 0.0 | 0.0 |
| *Pseudognaphalium affine* | 26.3 | 5.3 | 0.0 | 0.0 | 0.0 |
| *Rosa banksiae* | 0.0 | 0.0 | 0.0 | 10.0 | 0.0 |
| *Rosa x damascena;Rosa moschata;Rosa chinensis;Rosa dumalis* | 0.0 | 0.0 | 0.0 | 10.0 | 0.0 |
| *Rubus sp. MS-2014k* | 0.0 | 57.9 | 0.0 | 25.0 | 10.0 |
| *Rubus swinhoei* | 0.0 | 10.5 | 0.0 | 0.0 | 5.0 |
| *Rytidosperma auriculatum* | 31.6 | 0.0 | 0.0 | 0.0 | 0.0 |
| *Rytidosperma caespitosum* | 47.4 | 0.0 | 0.0 | 0.0 | 10.0 |
| *Rytidosperma clelandii* | 36.8 | 0.0 | 7.1 | 0.0 | 0.0 |
| *Rytidosperma gracile* | 57.9 | 5.3 | 35.7 | 0.0 | 0.0 |
| *Rytidosperma longifolium* | 47.4 | 5.3 | 0.0 | 5.0 | 0.0 |
| *Rytidosperma merum* | 31.6 | 0.0 | 0.0 | 0.0 | 0.0 |
| *Rytidosperma penicillatum* | 78.9 | 0.0 | 14.3 | 0.0 | 0.0 |
| *Rytidosperma pictum* | 26.3 | 5.3 | 35.7 | 0.0 | 10.0 |
| *Rytidosperma pulchrum* | 47.4 | 5.3 | 0.0 | 0.0 | 5.0 |
| *Rytidosperma racemosum* | 100.0 | 26.3 | 71.4 | 10.0 | 45.0 |
| *Rytidosperma sp. Humphreys 104* | 57.9 | 21.1 | 28.6 | 0.0 | 5.0 |
| *Rytidosperma telmaticum* | 10.5 | 0.0 | 0.0 | 0.0 | 0.0 |
| *Setaria parviflora* | 42.1 | 47.4 | 21.4 | 15.0 | 45.0 |
| *Setaria pumila* | 84.2 | 52.6 | 64.3 | 55.0 | 85.0 |
| *Silene gallica* | 0.0 | 0.0 | 0.0 | 10.0 | 5.0 |
| *Sinapis arvensis* | 0.0 | 0.0 | 14.3 | 0.0 | 0.0 |
| *Solenogyne dominii* | 21.1 | 0.0 | 0.0 | 0.0 | 0.0 |
| *Soliva sessilis* | 5.3 | 0.0 | 0.0 | 0.0 | 0.0 |
| *Sonchus oleraceus* | 5.3 | 15.8 | 0.0 | 0.0 | 0.0 |
| *Sporobolus fertilis* | 36.8 | 10.5 | 57.1 | 5.0 | 5.0 |
| *Stellaria longifolia* | 89.5 | 5.3 | 0.0 | 0.0 | 0.0 |
| *Stellaria pallida* | 10.5 | 15.8 | 7.1 | 15.0 | 5.0 |
| *Symphyotrichum subulatum* | 36.8 | 10.5 | 71.4 | 20.0 | 0.0 |
| *Symphyotrichum subulatum var. squamatum* | 5.3 | 0.0 | 14.3 | 0.0 | 0.0 |
| *Tagetes minuta* | 0.0 | 5.3 | 0.0 | 0.0 | 0.0 |
| *Taraxacum officinale* | 5.3 | 21.1 | 0.0 | 45.0 | 10.0 |
| *Themeda triandra* | 0.0 | 10.5 | 0.0 | 0.0 | 20.0 |
| *Thinopyrum elongatum* | 68.4 | 0.0 | 0.0 | 0.0 | 35.0 |
| *Tragus andicola* | 0.0 | 0.0 | 42.9 | 5.0 | 0.0 |
| *Tribulus terrestris* | 0.0 | 0.0 | 21.4 | 0.0 | 0.0 |
| *Trifolium arvense* | 36.8 | 0.0 | 0.0 | 0.0 | 5.0 |
| *Trifolium aureum* | 36.8 | 0.0 | 0.0 | 15.0 | 0.0 |
| *Trifolium* spp. | 42.1 | 21.1 | 50.0 | 100.0 | 15.0 |
| *Trifolium glomeratum* | 57.9 | 5.3 | 7.1 | 25.0 | 0.0 |
| *Trifolium nigrescens subsp. petrisavii* | 47.4 | 0.0 | 7.1 | 30.0 | 0.0 |
| *Trifolium repens* | 5.3 | 10.5 | 21.4 | 50.0 | 0.0 |
| *Triodia scariosa* | 0.0 | 0.0 | 21.4 | 0.0 | 0.0 |
| *Triticum turgidum subsp. durum* | 10.5 | 0.0 | 0.0 | 0.0 | 0.0 |
| Unassigned spp. | 100.0 | 100.0 | 85.7 | 100.0 | 70.0 |
| *Urochloa trichopus* | 15.8 | 15.8 | 92.9 | 35.0 | 5.0 |
| *Veronica anagallis-aquatica* | 0.0 | 10.5 | 7.1 | 0.0 | 0.0 |
| *Veronica arvensis* | 5.3 | 0.0 | 0.0 | 5.0 | 0.0 |
| *Vicia nigricans subsp. gigantea* | 15.8 | 26.3 | 0.0 | 5.0 | 0.0 |

Table S3. Pairwise SIMPER results showing the two taxa with the greatest contribution to dissimilarity for significant pairwise ANOSIM tests across the study sites

| Comparison | Badger Ground vs. Coolagolite | | Badger Ground vs. Merriwa | | Badger Ground vs. Robertson | | Badger Ground vs. Wolgan Valley | | Coolagolite vs. Merriwa | | Coolagolite vs. Robertson | | Coolagolite vs. Wolgan Valley | | Merriwa vs. Robertson | | Merriwa vs. Wolgan Valley | | Robertson vs. Wolgan Valley | |
| --- | --- | --- | --- | --- | --- | --- | --- | --- | --- | --- | --- | --- | --- | --- | --- | --- | --- | --- | --- | --- |
| Pairwise test: Av. Diss | 87.66 | | 91.25 | | 96.51 | | 84.41 | | 81.37 | | 87.61 | | 65.62 | | 96.16 | | 84.07 | | 91.62 | |
| Taxa | Av. Diss. | Cont (%) | Av. Diss. | Cont (%) | Av. Diss. | Cont (%) | Av. Diss. | Cont (%) | Av. Diss. | Cont (%) | Av. Diss. | Cont (%) | Av. Diss. | Cont (%) | Av. Diss. | Cont (%) | Av. Diss. | Cont (%) | Av. Diss. | Cont (%), |
| *Cynodon dactylon* | 11.5 | 13.12 |  |  |  |  | 9 | 10.66 | 9.65 | 11.86 | 11.74 | 13.41 | 9.13 | 13.91 |  |  | 9.74 | 11.58 |  |  |
| *Dactylis glomerata* |  |  |  |  | 23.14 | 23.97 |  |  |  |  | 22.53 | 25.72 |  |  | 22.35 | 23.24 |  |  | 21.65 | 23.63 |
| *Holcus lanatus* |  |  |  |  | 6.92 | 7.17 |  |  |  |  |  |  |  |  |  |  |  |  |  |  |
| *Paspalum dilatatum* | 11.73 | 13.38 | 6.01 | 6.58 |  |  | 15.82 | 18.74 | 11.66 | 14.33 |  |  | 15.57 | 23.73 |  |  | 16.57 | 19.71 | 17.68 | 19.29 |
| *Paspalidium constrictum* |  |  | 10.39 | 11.39 |  |  |  |  |  |  |  |  |  |  | 10.21 | 10.62 |  |  |  |  |

Av. Diss. = average dissimilarity; Cont (%) = contribution

Table S4. Pairwise SIMPER results showing the 10 taxa with the greatest contribution to dissimilarity for significant pairwise ANOSIM tests from Badger Ground

| Comparison | Autumn vs. winter | | Autumn vs. spring | | Autumn vs. summer | | Winter vs. spring | | Winter vs. summer | | Summer vs. spring | |
| --- | --- | --- | --- | --- | --- | --- | --- | --- | --- | --- | --- | --- |
| Pairwise test: Av. Diss | 89.74 | | 78.25 | | 61.18 | | 86.35 | | 89.65 | | 69.88 | |
| Taxa | Av. Diss. | Cont (%) | Av. Diss. | Cont (%) | Av. Diss. | Cont (%) | Av. Diss. | Cont (%) | Av. Diss. | Cont (%) | Av. Diss. | Cont (%), |
| *Panicum pauciflorum* | 10.61 | 11.82 | 8.01 | 10.24 | 6.98 | 11.41 | 5.18 | 6 | 7.24 | 8.08 | 3.39 | 4.85 |
| *Paspalum dilatatum* | 9.9 | 11.03 | 8.51 | 10.87 | 8.46 | 13.83 | 5.19 | 6.01 | 6.07 | 6.77 | 3.6 | 5.15 |
| *Rytidosperma racemosum* | 6 | 6.68 | 3.78 | 4.83 |  |  | 5.02 | 5.81 | 6.31 | 7.04 | 4.05 | 5.79 |
| *Pomax umbellata* | 5.86 | 6.53 |  |  |  |  | 5.86 | 6.79 | 5.86 | 6.54 |  |  |
| *Ectrosia schultzii* | 5.67 | 6.31 | 5.75 | 7.34 | 6.61 | 10.81 | 5.04 | 5.84 | 8.1 | 90.4 | 4.28 | 6.13 |
| *Medicago falcata* | 4.43 | 4.94 |  |  |  |  | 4.43 | 5.13 | 4.43 | 4.94 |  |  |
| *Cynodon dactylon* | 3.9 | 4.35 | 3.89 | 4.97 | 3.89 | 6.36 |  |  |  |  |  |  |
| *Paronychia baldwinii* | 3.64 | 4.06 | 4.11 | 5.26 | 3.55 | 5.8 |  |  |  |  |  |  |
| *Stellaria longifolia* | 3.24 | 3.61 |  |  |  |  | 3.25 | 3.76 | 3.25 | 3.62 |  |  |
| *Dichondra repens* | 3.11 | 3.47 |  |  |  |  |  |  |  |  |  |  |
| *Eragrostis tenuifolia* |  |  |  |  | 6.78 | 11.08 |  |  | 8.18 | 9.13 |  |  |
| *Digitaria cognata* |  |  | 2.58 | 3.29 | 2.87 | 4.7 |  |  | 3.52 | 3.93 | 3.4 | 4.86 |
| *Eragrostis tenuifolia* |  |  |  |  |  |  |  |  |  |  | 6.88 | 9.85 |
| *Eleusine tristachya* |  |  |  |  | 2.82 | 4.6 |  |  |  |  |  |  |
| Unassigned spp. |  |  |  |  | 2.53 | 4.13 |  |  |  |  |  |  |
| *Chloris gayana* |  |  |  |  |  |  |  |  | 3.17 | 3.53 |  |  |
| *Festuca bromoides* |  |  | 7.1 | 9.07 |  |  | 7.1 | 8.23 |  |  | 7.17 | 10.26 |
| *Hypochaeris* spp. |  |  | 6.35 | 8.12 |  |  | 5.92 | 6.85 |  |  | 5.95 | 8.52 |
| *Phalaris aquatica* |  |  | 4.02 | 5.14 |  |  | 5.59 | 6.47 |  |  | 4.02 | 5.76 |
| *Digitaria setigera* |  |  |  |  | 2.04 | 3.34 |  |  |  |  | 2.2 | 3.16 |

Av. Diss. = average dissimilarity; Cont (%) = contribution

Table S5. Pairwise SIMPER results showing the 10 taxa with the greatest contribution to dissimilarity for significant pairwise ANOSIM tests from Coolagolite

| Comparison | Autumn vs. winter | | Autumn vs. spring | | Autumn vs. summer | | Winter vs. spring | | Winter vs. summer | | Summer vs. spring | |
| --- | --- | --- | --- | --- | --- | --- | --- | --- | --- | --- | --- | --- |
| Pairwise test: Av. Diss | 60.78 | | 72.48 | | 38.82 | | 69.08 | | 57.66 | | 70.98 | |
| Taxa | Av. Diss. | Cont (%) | Av. Diss. | Cont (%) | Av. Diss. | Cont (%) | Av. Diss. | Cont (%) | Av. Diss. | Cont (%) | Av. Diss. | Cont (%) |
| *Paspalum dilatatum* | 20.39 | 33.56 | 22.61 | 31.2 | 12.36 | 31.83 | 6.59 | 9.55 | 14.45 | 25.06 | 19.79 | 25.32 |
| *Bromus racemosus* |  |  | 14.12 | 19.48 |  |  | 14.12 | 20.43 |  |  | 14.13 | 19.9 |
| *Cynodon dactylon* | 7.48 | 12.3 | 8.11 | 11.19 | 10.07 | 25.94 | 6.34 | 9.18 | 9.23 | 16.01 | 10.76 | 15.15 |
| *Holcus lanatus* | 5.18 | 8.53 | 4.06 | 5.6 | 3.87 | 9.97 | 3.48 | 5.04 | 3.35 | 5.81 | 1.33 | 1.88 |
| *Arrhenatherum elatius* | 5.41 | 8.9 | 4.03 | 5.56 | 2.99 | 7.69 | 4.11 | 5.96 | 5.56 | 9.65 | 3.32 | 4.68 |
| *Rubus sp. MS-2014k* |  |  | 3.96 | 5.46 |  |  | 3.95 | 5.72 |  |  | 3.95 | 5.57 |
| *Festuca bromoides* |  |  | 2.94 | 4.05 |  |  | 2.93 | 4.25 |  |  | 2.94 | 4.14 |
| *Entolasia stricta* | 1.31 | 2.16 | 1.86 | 2.56 |  |  |  |  |  |  | 1.84 | 2.59 |
| *Plantago lanceolata* |  |  | 0.93 | 1.29 |  |  |  |  |  |  |  |  |
| *Ludwigia peploides* |  |  | 0.89 | 1.22 |  |  |  |  |  |  |  |  |
| *Agrostis mertensii* |  |  |  |  | 0.51 | 1.32 |  |  |  |  |  |  |
| *Anthoxanthum aristatum* |  |  |  |  | 0.91 | 2.35 |  |  |  |  |  |  |
| *Axonopus polystachyus* | 1.99 | 3.27 |  |  | 1.21 | 3.13 |  |  | 2.04 | 3.54 | 1.27 | 1.8 |
| *Bromus catharticus* | 3.62 | 5.96 |  |  |  |  | 3.57 | 5.17 | 3.6 | 6.25 |  |  |
| *Dactylis glomerata* | 1.36 | 2.24 |  |  |  |  |  |  | 1.31 | 2.28 |  |  |
| *Eragrostis curvula* |  |  |  |  | 2.42 | 6.23 |  |  | 2.64 | 4.58 | 2.42 | 3.4 |
| *Glycine stenophita* |  |  |  |  | 0.51 | 1.31 |  |  |  |  |  |  |
| *Oplismenus undulatifolius* | 5.26 | 8.65 |  |  |  |  | 5.29 | 7.65 | 5.29 | 9.18 |  |  |
| *Phalaris arundinacea var. arundinacea* | 3.15 | 5.18 |  |  |  |  | 3.29 | 4.76 | 3.15 | 5.46 |  |  |
| *Setaria parviflora* |  |  |  |  | 0.51 | 1.31 |  |  |  |  |  |  |

Av. Diss. = average dissimilarity; Cont (%) = contribution

Table S6. Pairwise SIMPER results showing the 10 taxa with the greatest contribution to dissimilarity for significant pairwise ANOSIM tests from Merriwa

| Comparison | Autumn vs. winter | | Autumn vs. spring | | Autumn vs. summer | | Winter vs. spring | | Winter vs. summer | | Summer vs. spring | |
| --- | --- | --- | --- | --- | --- | --- | --- | --- | --- | --- | --- | --- |
| Pairwise test: Av. Diss | 85.79 | | 75.27 | | 79.79 | | 87.14 | | 93.8 | | 78.28 | |
| Taxa | Av. Diss. | Cont (%), | Av. Diss. | Cont (%), | Av. Diss. | Cont (%), | Av. Diss. | Cont (%), | Av. Diss. | Cont (%), | Av. Diss. | Cont (%), |
| *Paspalidium constrictum* | 22.76 | 26.53 | 2.68 | 3.56 | 3.82 | 4.79 | 24.22 | 27.79 | 25.46 | 27.15 |  |  |
| *Cynodon dactylon* | 2.99 | 3.48 | 2.01 | 2.67 | 8.66 | 10.85 | 2.7 | 3.09 | 11.23 | 11.97 | 8.71 | 11.13 |
| *Hymenachne grumosa* |  |  | 2.05 | 2.73 | 9.7 | 12.15 | 2.37 | 2.72 | 10.3 | 10.98 | 8.35 | 10.66 |
| *Chondrilla latifolia* | 8.86 | 10.32 |  |  |  |  | 8.66 | 9.94 | 8.86 | 9.44 |  |  |
| *Malva verticillata* |  |  |  |  | 4.23 | 5.3 |  |  | 4.31 | 4.59 | 4.26 | 5.44 |
| *Urochloa trichopus* |  |  |  |  | 4.17 | 5.22 |  |  | 4.29 | 4.57 | 4.22 | 5.39 |
| *Paspalum dilatatum* | 18.34 | 21.38 | 18.55 | 24.65 | 17.29 | 21.67 |  |  | 3.76 | 4.01 | 3.39 | 4.33 |
| *Glycine stenophita* |  |  |  |  | 3.45 | 4.33 |  |  | 3.47 | 3.7 | 3.47 | 4.44 |
| *Chloris truncata* | 3.08 | 3.59 | 6.42 | 8.53 |  |  | 6.51 | 7.47 | 3.21 | 3.42 | 6.05 | 7.73 |
| *Paspalum distichum var. indutum* |  |  |  |  |  |  |  |  | 1.92 | 2.04 |  |  |
| *Lolium perenne* |  |  | 3.77 | 5.01 |  |  | 3.77 | 4.32 |  |  | 3.77 | 4.82 |
| Unassigned spp. |  |  | 3.84 | 5.1 | 3.79 | 4.75 |  |  |  |  |  |  |
| *Hordeum murinum subsp. glaucum* |  |  | 5.32 | 7.06 |  |  | 5.31 | 6.1 |  |  | 5.32 | 6.79 |
| *Cynodon hirsutus* | 6.52 | 7.6 | 6.39 | 8.48 | 6.35 | 7.96 | 2.4 | 2.76 |  |  |  |  |
| *Austrostipa scabra* | 4.15 | 4.84 | 8.62 | 11.45 | 4.03 | 5.06 | 9.82 | 11.27 |  |  | 9.54 | 12.19 |
| *Eleusine tristachya* |  |  |  |  |  |  | 1.97 | 2.26 |  |  |  |  |
| *Entolasia stricta* | 1.87 | 2.19 |  |  |  |  |  |  |  |  |  |  |
| *Eucalyptus thozetiana* | 1.47 | 1.71 |  |  |  |  |  |  |  |  |  |  |
| *Enteropogon ramosus* | 1.46 | 1.7 |  |  |  |  |  |  |  |  |  |  |

Av. Diss. = average dissimilarity; Cont (%) = contribution

Table S7. Pairwise SIMPER results showing the 10 taxa with the greatest contribution to dissimilarity for significant pairwise ANOSIM tests from Robertson

| Comparison | Autumn vs. winter | | Autumn vs. spring | | Autumn vs. summer | | Winter vs. spring | | Winter vs. summer | | Summer vs. spring | |
| --- | --- | --- | --- | --- | --- | --- | --- | --- | --- | --- | --- | --- |
| Pairwise test: Av. Diss. | 49.21 | | 46.21 | | 65.15 | | 37.85 | | 68.86 | | 69.35 | |
| Taxa | Av. Diss. | Cont (%) | Av. Diss. | Cont (%) | Av. Diss. | Cont (%) | Av. Diss. | Cont (%) | Av. Diss. | Cont (%) | Av. Diss. | Cont (%) |
| *Dactylis glomerata* | 9.88 | 20.07 | 10.69 | 23.14 | 12.91 | 19.82 | 11.21 | 29.63 | 13.85 | 20.11 | 16.41 | 23.67 |
| *Paspalum dilatatum* | 2.11 | 4.28 | 1.88 | 4.08 | 13.27 | 20.36 | 2.06 | 5.45 | 13.09 | 19.01 | 13.53 | 19.51 |
| *Holcus lanatus* | 5.91 | 12.01 | 5.27 | 11.41 | 2.55 | 3.91 | 3.88 | 10.24 | 7.08 | 10.28 | 6.32 | 9.12 |
| *Lolium perenne* | 7.8 | 15.84 | 5.18 | 11.22 |  |  | 8.88 | 23.46 | 7.89 | 11.45 | 4.76 | 6.86 |
| *Hypochaeris* spp. | 1.49 | 3.03 | 1.49 | 3.23 | 3.84 | 5.9 |  |  | 4.13 | 6 | 4.12 | 5.95 |
| *Oenothera lindheimeri* |  |  |  |  | 4.1 | 6.3 |  |  | 4.1 | 5.96 | 4.1 | 5.91 |
| *Trifolium* spp. |  |  |  |  | 3 | 4.61 |  |  | 3.27 | 4.74 | 3.25 | 4.69 |
| *Malva verticillata* |  |  |  |  | 2.26 | 3.46 |  |  | 2.26 | 3.28 | 2.26 | 3.25 |
| *Entolasia stricta* | 8.29 | 16.85 | 8.25 | 17.84 | 8.13 | 12.49 | 0.79 | 2.09 | 1.91 | 2.77 | 1.99 | 2.88 |
| *Anthoxanthum aristatum* | 1.54 | 3.14 | 1.81 | 3.91 | 1.73 | 2.66 | 1.44 | 3.82 |  |  | 1.49 | 2.15 |
| *Poa annua* |  |  |  |  |  |  | 0.59 | 1.57 |  |  |  |  |
| *Ornithopus pinnatus* |  |  |  |  |  |  | 0.9 | 2.39 |  |  |  |  |
| *Bromus catharticus* |  |  |  |  |  |  | 1.12 | 2.95 |  |  |  |  |
| *Anthoxanthum sp. PT-2016* | 1.3 | 2.64 | 1.43 | 3.09 |  |  | 1.38 | 3.65 |  |  |  |  |
| *Panicum repens* | 1.6 | 3.25 | 1.6 | 3.46 |  |  |  |  |  |  |  |  |
| *Oplismenus undulatifolius* | 1.72 | 3.5 | 1.73 | 3.75 | 1.72 | 2.63 |  |  |  |  |  |  |
| *Axonopus polystachyus* |  |  |  |  |  |  |  |  | 1.34 | 1.95 |  |  |

Av. Diss. = average dissimilarity; Cont (%) = contribution
